# Ingested histamine and serotonin interact to alter *Anopheles stephensi* feeding and flight behavior and infection with *Plasmodium* parasites

**DOI:** 10.3389/fphys.2023.1247316

**Published:** 2023-07-24

**Authors:** Taylor A. Coles, Anna M. Briggs, Malayna G. Hambly, Nora Céspedes, Abigail M. Fellows, Hannah L. Kaylor, Alexandria D. Adams, Grace Van Susteren, Ronald E. Bentil, Michael A. Robert, Jeffrey A. Riffell, Edwin E. Lewis, Shirley Luckhart

**Affiliations:** ^1^ Department of Entomology, Plant Pathology, and Nematology, University of Idaho, Moscow, ID, United States; ^2^ Department of Biology, University of Washington, Seattle, WA, United States; ^3^ Department of Mathematics, Center for Emerging, Zoonotic, and Arthropod-Borne Pathogens (CeZAP), Virginia Tech, Blacksburg, VA, United States; ^4^ Department of Biological Sciences, University of Idaho, Moscow, ID, United States

**Keywords:** histamine, serotonin, *Anopheles stephensi*, flight behavior, feeding behavior, lifespan, *Plasmodium yoelii*, malaria

## Abstract

Blood levels of histamine and serotonin (5-HT) are altered in human malaria, and, at these levels, we have shown they have broad, independent effects on *Anopheles stephensi* following ingestion by this invasive mosquito. Given that histamine and 5-HT are ingested together under natural conditions and that histaminergic and serotonergic signaling are networked in other organisms, we examined effects of combinations of these biogenic amines provisioned to *A. stephensi* at healthy human levels (high 5-HT, low histamine) or levels associated with severe malaria (low 5-HT, high histamine). Treatments were delivered in water (priming) before feeding *A. stephensi* on *Plasmodium yoelii*-infected mice or via artificial blood meal. Relative to effects of histamine and 5-HT alone, effects of biogenic amine combinations were complex. Biogenic amine treatments had the greatest impact on the first oviposition cycle, with high histamine moderating low 5-HT effects in combination. In contrast, clutch sizes were similar across combination and individual treatments. While high histamine alone increased uninfected *A. stephensi* weekly lifetime blood feeding, neither combination altered this tendency relative to controls. The tendency to re-feed 2 weeks after the first blood meal was altered by combination treatments, but this depended on mode of delivery. For blood delivery, malaria-associated treatments yielded higher percentages of fed females relative to healthy-associated treatments, but the converse was true for priming. Female mosquitoes treated with the malaria-associated combination exhibited enhanced flight behavior and object inspection relative to controls and healthy combination treatment. Mosquitoes primed with the malaria-associated combination exhibited higher mean oocysts and sporozoite infection prevalence relative to the healthy combination, with high histamine having a dominant effect on these patterns. Compared with uninfected *A. stephensi*, the tendency of infected mosquitoes to take a second blood meal revealed an interaction of biogenic amines with infection. We used a mathematical model to project the impacts of different levels of biogenic amines and associated changes on outbreaks in human populations. While not all outbreak parameters were impacted the same, the sum of effects suggests that histamine and 5-HT alter the likelihood of transmission by mosquitoes that feed on hosts with symptomatic malaria *versus* a healthy host.

## 1 Introduction

The 2022 World Malaria Report recorded 247 million cases in 2021, compared to 245 million cases in 2020, with an estimated 619,000 deaths in 2021. This increase in cases was attributed to resource and capacity disruptions due to COVID-19 (World Health Organization, 2022). Accordingly, malaria remains a global burden (Kumar, 2021) and a disease of significant public health importance (World Health Organization, 2022). Malaria pathology is complex and dependent on previous parasite exposure, age, other underlying conditions, parasite species, and host genetics, among the major factors. Like other parasitic infections, malaria has been associated with allergic inflammation, which is characterized by the activation of mast cells and basophils, the major cellular sources of the biogenic amine histamine, and a shift to a Th2-type immune response (Donnelly et al., 2021). Allergic inflammation has also been associated with reduced production of serotonin or 5-hydroxytryptamine (5-HT; Van der Leek et al., 2017). Taken together, it is perhaps not surprising that severe malaria has been associated with increased histamine and reduced 5-HT levels in blood (Enwonwu et al., 1999; Enwonwu et al., 2000; Lopansri et al., 2006).

Histidine decarboxylase (HDC) catalyzes the synthesis of histamine from histidine via decarboxylation (Epps, 1945). In mammals, histamine regulates the host immune response and inflammation, gastric acid secretion, and neurotransmission (Höcker et al., 1996). In severe malaria, circulating blood histamine levels can rise nearly 10-fold to 10 nM above healthy levels (Enwonwu et al., 2000; Lopansri et al., 2006). Notably, high histamine levels consistent with acute inflammation inhibit 5-HT release via histamine receptor activation at serotonergic neurons, indicating profound networking of these biogenic amines (Schlicker et al., 1988; Fink et al., 1990; Threlfell et al., 2004; Hersey et al., 2021; Hersey et al., 2022). 5-HT is synthesized from the amino acid tryptophan via tryptophan hydroxylase and translates information between nerve cells in the brain and other parts of the body (Gao et al., 2020). Blood 5-HT levels in healthy adults range from 0.6 µM to 1.5 µM, while in healthy children, they range from 0.4 µM to 2.6 µM. Serotonin levels as low as 0.1 µM have been linked to severe malaria (Loo, 1974; Badcock et al., 1987; Winn et al., 2018).

Anopheline mosquitoes transmit protozoan parasites of the genus *Plasmodium*, the causative agents of malaria (Tadesse et al., 2021). During blood feeding by a mosquito, factors in human blood, including biogenic amines, are ingested. Both histamine and 5-HT are important neuromodulators in insects, including hematophagous vectors. For example, 5-HT is strongly expressed in the peripheral and central nervous system of *Aedes aegypti*, *Anopheles gambiae*, and *Rhodnius prolixus*, with neural processes that connect the brain with peripheral tissues, including the gut, peripheral appendages, and muscles (Lange et al., 1988; Siju et al., 2008; Rodriguez et al., 2021). Serotonergic processes also densely innervate the mouthparts. These observations collectively affirm 5-HT modulation of sensory behavior during flight and feeding. Our previous work showed distinct innervation patterns of histamine and 5-HT in regions of the gut and brain of *Anopheles stephensi*, with effects of histamine and 5-HT on mosquito infection and behavior that were similar but not identical in promoting parasite transmission (Rodriguez et al., 2021; Briggs et al., 2022). Based on these observations and the facts that histamine and 5-HT are ingested together in blood by *A. stephensi* and histaminergic and serotonergic signaling are networked in other organisms (Haas et al., 2008), we sought to understand the combined effects of ingested histamine and 5-HT on *A. stephensi*, as well as how these effects may influence malaria dynamics at the population level. We incorporated the results of these experiments into a mathematical model to project how changes in mosquito behavior and physiology may impact transmission dynamics in host populations.

In this study, we examined the effects of ingested healthy- and malaria-associated levels of histamine and 5-HT on *A. stephensi* infection and behavior. Based on these observations, we also considered the potential effects of histamine and 5-HT on population-level malaria transmission by incorporating our findings into a mathematical model.

## 2 Materials and methods

### 2.1 Mosquito rearing

Indian wild-type strain *A. stephensi* were maintained as previously described (Rodriguez et al., 2021; Briggs et al., 2022). All mosquito life stages were maintained at 28°C, 80% humidity, and under 12 h light–dark cycles (0800–2000). At a minimum, 24 h prior to the start of an experiment, adult female mosquitoes were moved and housed in 2 L polypropylene containers with mesh screening.

### 2.2 Treatments

To test the impacts of ingested histamine and 5-HT in combination on *A. stephensi*, 4–6 day old adult female mosquitoes were provisioned with the following treatments delivered as described below: control (water only), malaria-associated histamine (10 nM histamine), malaria-associated 5-HT (0.15 μM 5-HT), healthy-associated histamine (1 nM histamine), healthy-associated 5-HT (1.5 µM 5-HT), malaria-associated combination (0.15 µM 5-HT + 10 nM histamine) and healthy-associated combination (1.5 µM 5-HT + 1 nM histamine).

### 2.3 Artificial blood meal delivery to *A. stephensi*

As described previously, (Rodriguez et al., 2021; Briggs et al., 2022), a blood meal consisting of washed human red blood cells (RBCs) and heat inactivated human serum (1:1, vol:vol) was provided via glass bell feeders to *A. stephensi* for 15 min. Time of blood feeding has been shown to alter some reproductive variables, but infection success is not impacted (O’Donnell et al., 2019), so blood meals were offered between 0800–1,100 to maintain consistency across all studies. Sucrose-soaked cotton balls (10%) were used to maintain mosquitoes between blood meals.

### 2.4 Provisioning of histamine and 5-HT via artificial blood meal to uninfected *A. stephensi*

The treatments described in Section 2.2 were provisioned via artificial blood meal (Section 2.3) to mosquitoes, after which experiments on behavior, reproduction, lifespan and infection (described below in Sections 2.8–2.13) were conducted. Histamine and 5-HT were diluted in water then diluted as 3 μL aliquots in 3 mL of blood for the final concentrations indicated in Section 2.2.

### 2.5 Provisioning of histamine and 5-HT to *A. stephensi* via priming

The treatments described in Section 2.2 were provisioned to mosquitoes by delivery of treatment-soaked cotton balls prior to the first blood meal, previously described as priming (Rodriguez et al., 2021; Briggs et al., 2022). Priming was used to provision treatments prior to feeding on infected mice. A subset of behavioral studies was completed with primed, uninfected *A. stephensi* for direct comparison to studies with primed, infected *A. stephensi*. Female mosquitoes received a sugar cube and treatment-soaked cotton balls with the treatments from Section 2.2. The treatment solutions were made fresh daily, and treatment-soaked cotton balls were changed twice per day between 0830–1,100 and 1,630–1800 for 3 days prior to the first blood meal. Prior to the first blood meal, the sugar cube and treatment-soaked cotton balls were removed within 30 min to 1 h.

### 2.6 Histamine and 5-HT provisioning to assess effects on reproduction of uninfected *A. stephensi*

Each treatment (Section 2.2) was offered in a blood meal to groups of 120 female mosquitoes as described in Section 2.4. Following feeding, 40 blood-fed mosquitoes from each treatment group were placed into individual housing (50 mL conical tubes) for 48 h to oviposit as described (Rodriguez et al., 2021; Briggs et al., 2022). Following oviposition, female mosquitoes were transferred back to group housing and eggs were counted and recorded. The process was repeated weekly for 3 weeks or three gonotrophic cycles with blood fed mosquitoes. Separate cohorts of mosquitoes were used to perform five biological replicates.

### 2.7 Histamine and 5-HT provisioning to assess effects on lifetime blood feeding and survival of uninfected *A. stephensi*

Each combination treatment (Section 2.2) was offered in a blood meal to groups of 120 female mosquitoes as described in Section 2.4. Mosquitoes were offered a treated blood meal once weekly until no mosquitoes remained alive as described (Rodriguez et al., 2021; Briggs et al., 2022). After the first blood meal only, non-fed mosquitoes were discarded. A 250 mL polypropylene cup lined with moistened filtration paper was provided for oviposition after each blood meal. Each week the numbers of blood fed and non-fed mosquitoes were counted and recorded. Separate cohorts of mosquitoes were used to perform three biological replicates.

### 2.8 Histamine and 5-HT provisioning to assess effects on tendency to take a second blood meal by uninfected *A. stephensi*

Female mosquitoes were treated as described in Section 2.4 with delivery of treatments in the first blood meal or as described in Section 2.5 with priming prior to the first blood meal (Rodriguez et al., 2021; Briggs et al., 2022). For these studies, 80–120 female mosquitoes were moved and housed in 2 L polypropylene containers with mesh screening. Following the first blood meal, non-fed mosquitoes were discarded. At 4 days or 14 days after the first blood meal, a second blood meal was offered, and blood fed and non-fed mosquitoes were recorded. Separate cohorts of mosquitoes were used for 4 days and 14 days feeding studies. Five biological replicates were performed for both priming and blood meal delivery of the treatments.

### 2.9 Histamine and 5-HT provisioning to assess flight behavior of uninfected *A. stephensi*

Flight behavior experiments were conducted in flight cages under ambient environmental conditions (<10 cm/s air velocities, 22.5°C and 410 ppm CO_2_) which allowed rapid testing of different treatment conditions. The flight assay used 30 cm × 30 cm × 30 cm cages (Bioquip), in a manner similar to the experiments by Rodriguez et al. (2021), which were conducted in a wind tunnel. Briefly, a Photonic Fence Monitoring Device (PFMD), a 3D insect tracking system (Photonic Sentry, Bellevue, WA), was used to track mosquito trajectories. The system is comprised of two cameras, each associated with a ring of infrared LEDs, positioned on the side of the cage to record mosquito trajectories at 100 frames/s. A retroreflective material (3M Scotchlite 8910) was installed at the back of the cages to provide backlighting of the flying mosquitoes. The ambient temperature and CO_2_ outside of the tunnel were 22.5°C and 410 ppm (ca. 0.041%), respectively. For each assay, approximately 25 female mosquitoes were transferred into the arena and allowed to acclimate for 15 min. In these experiments, filtered air was released into the cage for 20 min (pre-CO_2_), followed by 5% CO_2_ for 20 min, after which filtered air was returned to the cage for 20 min (post-CO_2_). The 5% CO_2_ plume or filtered air was delivered using two mass flow controllers (MC-200SCCM-D, Alicat Scientific, Tucson, AZ) controlled by a Python script that allowed synchronizing odor and filtered air delivery with the trajectory behaviors. To examine mosquito responses to visual stimuli, we randomly placed 3 cm diameter white and black paper circles (Color-aid Corp., Hudson Falls, NY, United States) 20 cm apart on the floor of the cage. The tracking system cannot maintain mosquito identities for extended periods of time, but individual trajectories were assumed to be independent for statistical analysis. Trajectories that were captured for at least 60 frames (1 s) were included in the analyses. Flight velocities for each mosquito trajectory were recorded and analyzed based on their 3D trajectory for the pre-CO_2_ (filtered air), and CO_2_ conditions. Attraction of mosquitoes to the visual cues was examined by summing the total number of mosquito trajectories that came within 3 cm of the cues. Experiments were performed with mosquitoes provisioned with histamine and 5-HT in combination at healthy and malaria-associated levels in an artificial blood meal (Section 2.4).

### 2.10 Infection of *A. stephensi* with *Plasmodium y. yoelii* 17XNL

The mouse malaria parasite *P. y. yoelii* 17XNL (hereafter, *P. yoelii*) was maintained in 9–11-week-old CD-1 mice (Envigo, Indianapolis, IN) as previously described (Rodriguez et al., 2021; Briggs et al., 2022). All procedures were approved by the Institutional Animal Care and Use Committee of the University of Idaho (protocol IACUC-2023-8, renewed 2/27/2023). Mice were infected by injection of *P. yoelii-*infected RBCs and monitored daily for parasitemia by Giemsa staining starting 2 days after infection. Exflagellation was evaluated using wet prep slides with a drop of blood at 3 days post-infection. A total of 4–10 fields were counted for exflagellation events to identify mice with similar exflagellation rates for mosquito infection. Mice were anesthetized using ketamine (50 mg/kg) and xylazine (5 mg/kg) in sterile saline and placed on top of 2 L polypropylene cartons with 100–120 female mosquitoes. Mosquitoes were allowed access to mice for 30 min, after which non-fed mosquitoes were removed and discarded. After mosquito feeding, mice were euthanized via CO_2_ asphyxiation followed by cervical dislocation.

### 2.11 Histamine and 5-HT provisioning to assess infection of *A. stephensi* with *P. yoelii*

For these studies, 100–120 female mosquitoes were housed in 2 L polypropylene containers with mesh screening. The mosquitoes were primed for 3 days prior to infection as described in Section 2.5, and then infected as described in Section 2.10. At 10 days post-feeding, midguts were dissected from 15 to 20 mosquitoes and stained with mercurochrome to count oocysts. At 14–15 days post-feeding, salivary glands were dissected and scored from 10 to 15 mosquitoes using the following scale: 1 for 1–99 sporozoites, 2 for 100–999 sporozoites, 3 for 1,000–9,999 sporozoites, and 4 for 10,000+ sporozoites. Oocyst and sporozoite infections were assessed with separate cohorts of mosquitoes with four biological replicates. Salivary gland infections were scored by the same person for all replicates.

### 2.12 Histamine and 5-HT provisioning to assess the tendency of *P. yoelii*-infected *A. stephensi* to take a second blood meal

Infected female mosquitoes from Section 2.11 were also used for feeding behavior studies. A cohort of 15–20 female mosquitoes from each treatment group was moved and housed in 0.95 L polypropylene containers with mesh screening and were provisioned a second blood meal at 4 days following infection. An additional cohort of 15–20 female mosquitoes was moved and provisioned a second blood meal at 11 days following infection. Each time point used different cohorts of mosquitoes where the numbers of blood fed and non-blood fed mosquitoes were recorded, and then discarded. Four biological replicates were performed.

### 2.13 Statistical analyses

Average numbers of eggs per female per gonotrophic cycle were analyzed using two-way analysis of variance (ANOVA). Main effects were biogenic amine treatment and gonotrophic cycle. Because both main effects and the interaction between them showed significant differences, means were separated using LS Means Differences Student’s t-test. The percentages of mosquitoes ovipositing were analyzed using a Chi-square test between treatments. Kaplan-Meier analysis followed by the log-rank test of Mantel-Cox and Gehan-Breslow-Wilcoxon were used to analyze the individual replicates of lifespan to compare the treatments, in both survivorship and the duration of feeding behavior. The median survival in days and the median duration of feeding in weeks were compared among treatments using the Kruskal-Wallis ANOVA for non-normal data. Means were separated using Dunn’s multiple comparisons test where appropriate. Percentages of uninfected and infected *A. stephensi* taking a second blood meal and infection prevalence of oocysts and sporozoites were analyzed using Chi-square to compare treatments. Oocyst and sporozoite infection intensities were analyzed using one-way ANOVA and means were separated using the Newman-Keuls multiple comparisons test. For flight assays, the mean activity index pre- and during- CO_2_, mosquito attraction to visual objects during CO_2_, and the mean velocity of mosquito trajectories during CO_2_ were compared among treatments using the Kruskal-Wallis test. Means were separated *post hoc* using Dunn’s multiple comparisons test.

### 2.14 Mathematical model

We constructed a mathematical model to project potential population level effects on human hosts of biogenic amines delivered by priming to uninfected mosquitoes. This scenario was selected to model the appearance of infection in exposed mosquitoes with different levels of biogenic amines and the associated potential changes to malaria outbreak parameters in human populations. Accordingly, the model is a host-vector epidemiological compartmental model comprised of discrete time equations. The general model considers a human population divided into compartments by infection status: Susceptible, Exposed, Infectious, and Recovered. The mosquito population is divided similarly: Susceptible, Exposed, Infectious. In both populations, susceptible individuals are not infected but can become infected. Exposed individuals have been infected but are not yet infectious; in this stage, the pathogen is undergoing an incubation period. Infectious individuals are infected and capable of transmitting the pathogen. Finally, humans enter a recovered class when they have cleared an infection. The mosquito population is divided by age classes: eggs, larvae and pupae, and adult classes from ages 1, 2, … N_A_, where N_A_ is the maximum number of age classes included in the model. Rates of transitions among the compartments were determined by parameters related to disease processes. To compare the impacts of biogenic amines, we used different parameterizations of the model. We considered three different scenarios with the model: biogenic amines impacting the tendency to take a second blood meal 4 and 14 days later, fecundity, or the combination of tendency to take a second blood meal and fecundity. We opted not to explore all experimental results in this work as this is planned for a future study with this model. We projected model outputs of human malaria such as daily incidence, time and size of the peak daily incidence, total cases, endemic prevalence, and daily prevalence. A full description of the model and parameters are included in Supplementary Material.

## 3 Results

### 3.1 Treatments with low *versus* high concentrations of histamine and 5-HT were associated with increased oviposition, with the effect of low 5-HT enhanced by high histamine

**FIGURE 1:**
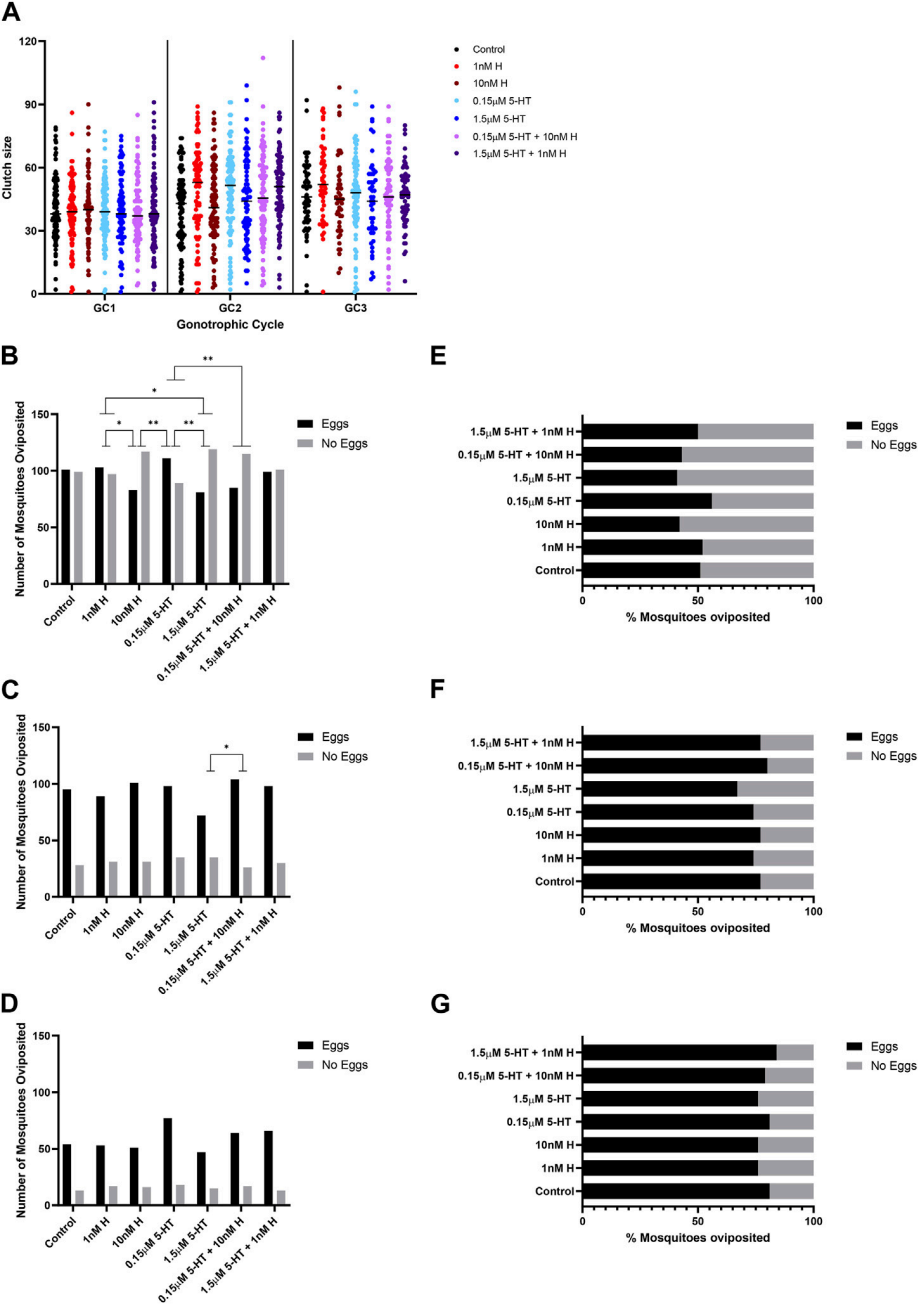
Oviposition of uninfected *A. stephensi* provisioned with blood supplemented with histamine and 5-HT alone and in combination. **(A)** The numbers of eggs laid per female *A. stephensi* in three gonotrophic cycles (GC). N = 5; two-way ANOVA (see Supplementary Table S1). The numbers of *A. stephensi* that oviposited after each gonotrophic cycle, **(B)** GC1, **(C)** GC2, **(D)** GC3. The percentages of *A. stephensi* that oviposited after each gonotrophic cycle, **(E)** GC1, **(F)** GC2, **(G)** GC3. N = 5; Chi-square analysis, *1 nM H vs. 10nM H *p* = 0.0450, *1 nM H vs. 1.5 μM 5-HT *p* = 0.0273, **10 nM H vs 0.15 μM 5-HT *p* = 0.0051, **0.15 μM 5-HT vs. 1.5 μM 5-HT *p* = 0.0027, **1.5 μM 5-HT vs. 0.15 μM 5-HT + 10 nM H *p* = 0.0093, **0.15 μM 5-HT vs. 0.15 μM 5-HT + 10 nM H *p* = 0.0093. H, histamine; 5-HT,serotonin.

A two-way ANOVA was performed to evaluate the effects of gonotrophic cycle and biogenic amine treatment on clutch size. Means and standard errors for all combinations are provided in Supplementary Table S1. There were significant main effects for both gonotrophic cycle [F (2,1711) = 28.82, *p* < 0.001] and biogenic amine treatment [F (6,1711) = 2.17, *p* = 0.043]. We also found a significant interaction between the two main effects [F (12,1731) = 1.81, *p* < 0.04]. Across all treatments, the average clutch size in the first gonotrophic cycle (39.65 ± 0.69) was significantly smaller than those in the second (45.74 ± 0.70) and third (47.04 ± 0.88) cycles. Across gonotrophic cycles, mosquitoes provisioned with low histamine produced significantly more eggs (44.66 ± 1.06) than control mosquitoes (41.63 ± 1.16) and more than mosquitoes treated with high 5-HT (42.44 ± 1.29) or high histamine (42.00 ± 1.20). The significant interaction between main effects showed that treatment with histamine and 5-HT have differing effects on patterns of egg production across gonotrophic cycles (Supplementary Table S1). Control mosquitoes and mosquitoes treated with high and low histamine peaked in clutch size in the third gonotrophic cycle whereas other treatments peaked during the second gonotrophic cycle (Supplementary Table S1). There were also significant differences in the percentages of females laying eggs in the first and second gonotrophic cycles, but not in the third gonotrophic cycle (Figures 1B–D for raw numbers, Figures 1E–G for corresponding percent data). In the first gonotrophic cycle, oviposition percentages were increased in groups treated with low histamine and low 5-HT compared to the respective high treatments (Figures 1B, E). The effect of low 5-HT was moderated by 10 nM histamine, the combination which represents malaria-associated levels of biogenic amines (Figures 1B, E). In the second gonotrophic cycle, the percentage of mosquitoes ovipositing was lowest in the group treated with high 5-HT and this was significantly different from the group treated with the malaria-associated combination of histamine and 5-HT (Figures 1C, F).

### 3.2 Treatment with histamine and 5-HT in combination had no effects on uninfected *A. stephensi* lifespan or lifetime blood feeding

**FIGURE 2:**
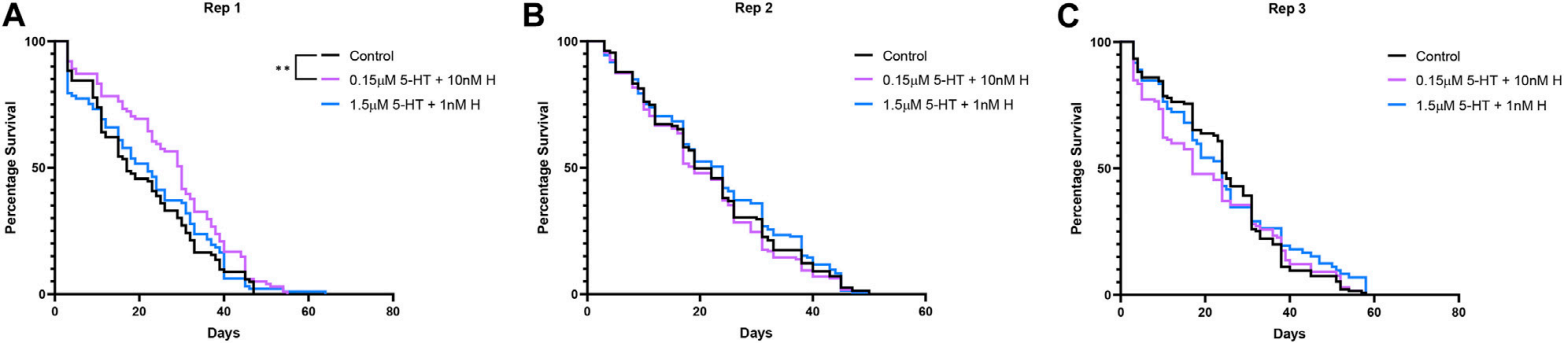
Survival of uninfected *A. stephensi* provisioned weekly blood meals supplemented with combinations of histamine and 5-HT or with an equivalent volume of added water (control). A simple survival analysis (Kaplan-Meier) was used to compare all the treatment groups within each replicate. **(A)** Replicate 1 (Rep 1), **control vs 0.15 µM 5-HT + 10 nM H *p* = 0.0036, **(B)** Replicate 2 (Rep 2), no significance, **(C)** Replicate 3 (Rep 3), no significance. H, histamine; 5-HT, serotonin.

**FIGURE 3:**
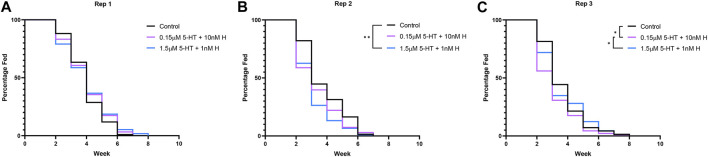
Blood feeding over lifespan of uninfected *A. stephensi* provisioned weekly blood meals supplemented with combinations of histamine and 5-HT or with an equivalent volume of added water (control). A simple survival analysis (Kaplan-Meier) was used to compare all the treatment groups within each replicate. **(A)** Replicate 1 (Rep 1), no significance, **(B)** Replicate 2 (Rep 2), **control vs 1.5 μM 5-HT + 1 nM H *p* = 0.0029, **(C)** Replicate 3 (Rep 3), *control vs 0.15 μM 5-HT + 10 nM H *p* = 0.0472, *0.15 μM 5-HT + 10 nM H vs 1.5 μM 5-HT + 1 nM H *p* = 0.0445. H, histamine; 5-HT, serotonin.

Treatment with the malaria-associated combination of histamine and 5-HT was associated with a significant increase in mosquito survival relative to control only in the first replicate (Figures 2A–C; Supplementary Table S2), but there were no treatment effects on median lifespan in any replicate (Supplementary Figure S1). Treatment had variable effects on the percentages of mosquitoes feeding weekly over lifespan in two of three replicates (Figures 3A–C; Supplementary Table S3), but there were no effects of treatment on the median week of blood feeding cessation for any replicate (Supplementary Figure S2).

### 3.3 The effects of histamine and 5-HT on the tendency of uninfected *A. stephensi* to take a second blood meal were dependent on delivery method, treatment and timing of the second meal

**FIGURE 4:**
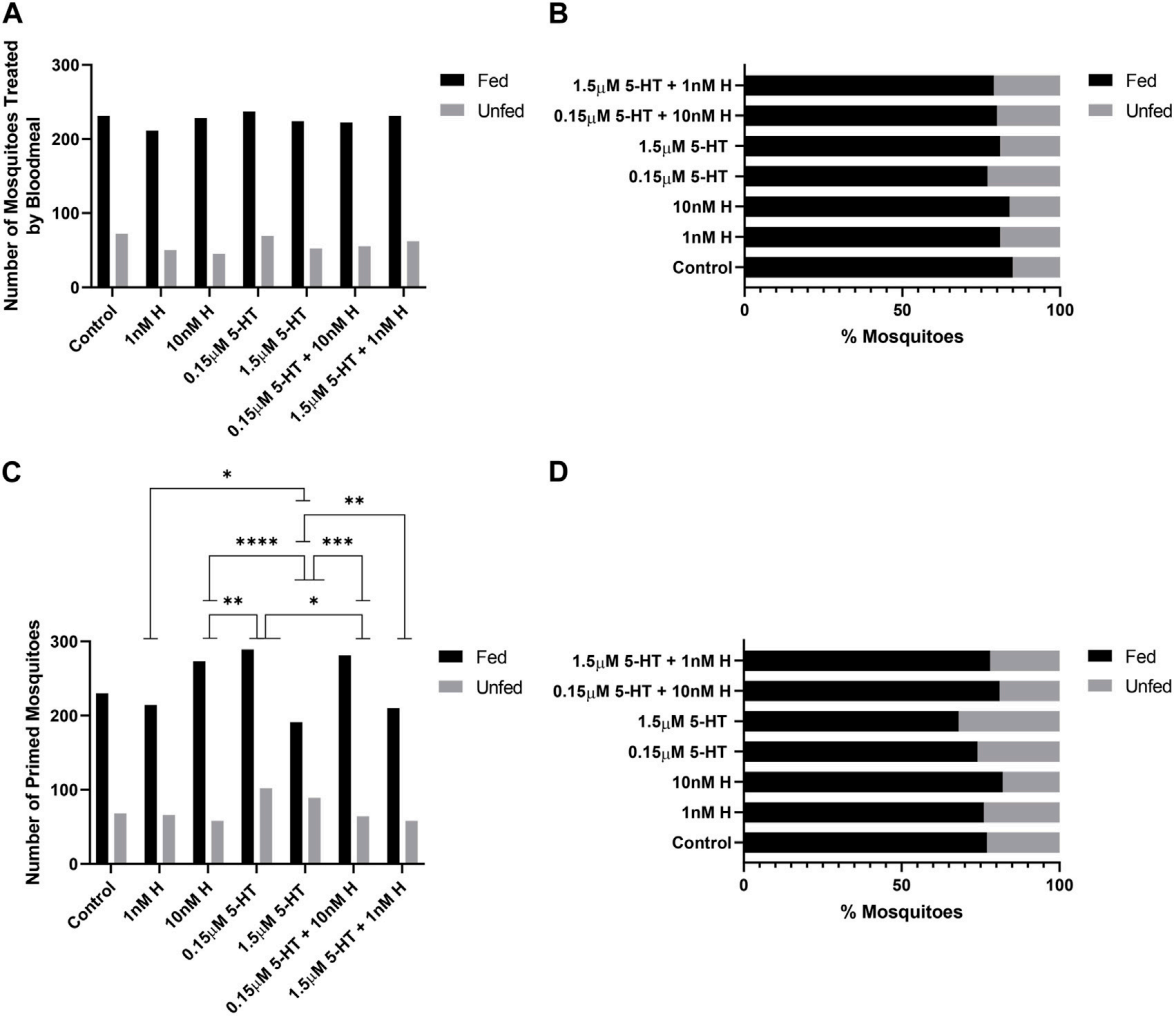
Tendency of uninfected *A. stephensi* to take a second blood meal 4 days later. **(A)** Numbers of fed and unfed mosquitoes provisioned with histamine and 5-HT treatments in the first blood meal. N = 5; Chi-square test (α = 0.05), no significance. **(B)** Percentages of fed and unfed mosquitoes provisioned with histamine and 5-HT treatments in the first blood meal. **(C)** Numbers of fed and unfed mosquitoes primed for 3 days with histamine and 5-HT treatments in water or with water-only soaked cotton balls. N = 5; Chi-square test (α = 0.05), *1 nM H vs 1.5 μM 5-HT *p* = 0.0298, **10 nM H vs. 0.15 μM 5-HT *p* = 0.0058, ****10 nM H vs. 1.5 μM 5-HT *p* = <0.0001, *0.15 μM 5-HT vs. 0.15 μM 5-HT + 10 nM H *p* = 0.0146, ***1.5 μM 5-ΗΤ vs. 0.15 μM 5-HT + 10 nM H *p* = 0.0001, **1.5 μM 5-HT vs. 1.5 μM 5-HT + 1 nM H *p* = 0.0074. **(D)** Percentages of fed and unfed mosquitoes primed with histamine and 5-HT treatments. H, histamine; 5-HT, serotonin.

**FIGURE 5:**
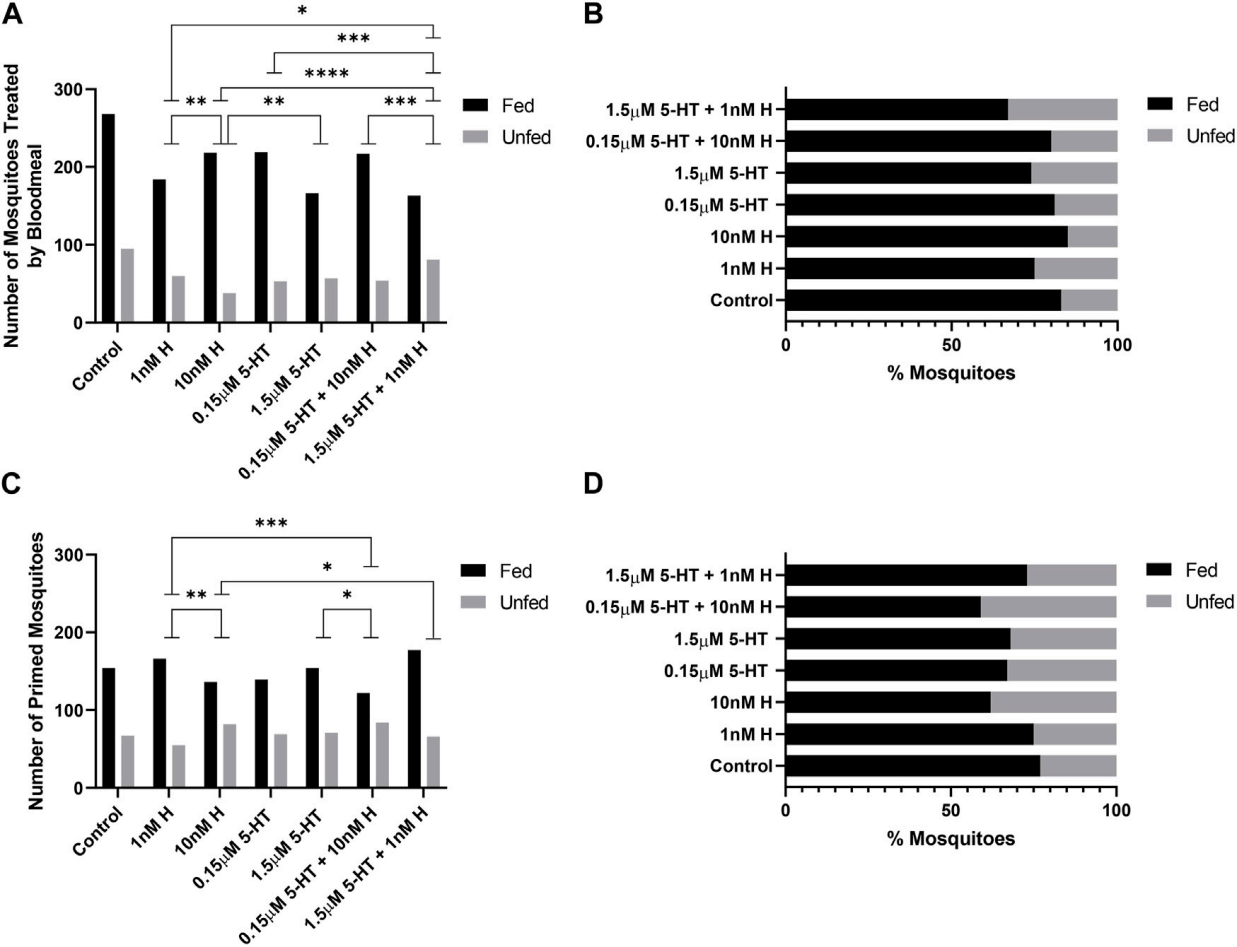
Tendency of uninfected *A. stephensi* to take a second blood meal 14 days later. **(A)** Numbers of fed and unfed mosquitoes provisioned with histamine and 5-HT treatments in the first blood meal. N = 5; Chi-square test (α = 0.05), **1 nM H vs. 10nM H *p* = 0.0061, *1 nM H vs. 1.5 μM 5-HT + 1 nM H *p* = 0.0360, **10 nM H vs. 1.5 μM 5-HT *p* = 0.0033, ****10 nM H vs. 1.5 μM 5-HT + 1 nM H *p* = <0.0001, ***0.15 μM 5-HT vs. 1.5 μM 5-HT + 1 nM H *p* = 0.0004, ***0.15 μM 5-HT + 10 nM H vs. 1.5 μM 5-HT + 1 nM H *p* = 0.0006. **(B)** Percentages of fed and unfed mosquitoes provisioned with histamine and 5-HT treatments in the first blood meal. **(C)** Numbers of fed and unfed mosquitoes following priming for 3 days with histamine and 5-HT treatments in water or with water-only soaked cotton balls. N = 5; Chi-square test (α = 0.05), **1 nM H vs. 10nM H *p* = 0.0040, ***1 nM H vs. 0.15 μM 5-HT + 10 nM H *p* = 0.0005, *10 nM H vs. 1.5 μM 5-HT + 1 nM H *p* = 0.0164, *1.5 μM 5-HT vs. 0.15 μM 5-HT + 10 nM H *p* = 0.0463. **(D)** Percentages of fed and unfed mosquitoes primed with histamine and 5-HT treatments. H, histamine; 5-HT, serotonin.

In previous studies, provisioning of malaria-associated histamine in blood was associated with increased percentages of uninfected *A. stephensi* taking a second blood meal at both 4 days and 14 days, while there were no effects of histamine delivered by priming at either time point (Rodriguez et al., 2021). Conversely, provisioning of healthy-associated 5-HT by priming and blood was associated with increased percentages of uninfected *A. stephensi* taking a second blood meal at 4 days, but there were no effects of 5-HT delivered by priming or blood on tendency to take a second blood meal at 14 days (Briggs et al., 2022). Based on these observations, we sought to directly compare treatments with histamine and 5-HT with the effects of malaria-associated and healthy-associated combinations delivered via blood or priming on the tendency of uninfected *A. stephensi* to take second blood meals at 4 days (Figures 4A–D) and 14 days (Figures 5A–D).

At 4 days, treatment effects on the tendency to take a second blood meal were observed only for mosquitoes provisioned by priming (Figures 4C, D). Histamine treatment was associated with increased feeding tendency relative to 5-HT treatment (1 nM histamine vs. 1.5 µM 5-HT, 10 nM histamine vs. 1.5 µM 5-HT, 10 nM histamine vs. 0.15 µM 5-HT; *p* < 0.05). Further, the addition of malaria-associated or healthy-associated histamine to 5-HT increased the tendency to feed relative to the same concentration of 5-HT (0.15 µM 5-HT vs. 0.15 µM 5-HT + 10 nM histamine, 1.5 µM 5-HT vs. 1.5 µM 5-HT + 1 nM histamine; *p* < 0.05). Hence, at 4 days, the tendency to take a second blood was enhanced by histamine, but this effect was limited to provisioning by priming.

At 14 days, treatment effects on the tendency to take a second blood meal were observed for mosquitoes provisioned with histamine and 5-HT in blood (Figures 5A, B) and by priming (Figures 5C, D). The effects of provisioning by priming and blood, however, were distinct. Specifically, for provisioning in blood, in each case where there was a difference between malaria-associated and healthy-associated treatments (1 nM vs. 10 nM histamine, 10 nM histamine vs. 1.5 µM 5-HT, 10 nM histamine vs. 1.5 µM 5-HT + 1 nM histamine, 0.15 µM 5-HT vs. 1.5 µM 5-HT + 1 nM histamine, 0.15 µM 5-HT + 10 nM histamine vs. 1.5 µM 5-HT + 1 nM histamine; *p* < 0.05), malaria-associated treatments yielded higher percentages of fed females relative to healthy-associated treatments (Figures 5A, B). For priming, in each case where there was a difference between malaria-associated and healthy-associated treatments (1 nM vs. 10 nM histamine, 1 nM histamine vs. 0.15 µM 5-HT + 10 nM histamine, 10 nM histamine vs. 1.5 µM 5-HT + 1 nM histamine, 1.5 µM 5-HT vs. 0.15 µM 5-HT + 10 nM histamine; *p* < 0.05), malaria-associated treatments yielded lower percentages of fed females relative to healthy-associated treatments (Figures 5C, D). Similarly, the percentage of fed mosquitoes treated with the malaria-associated combination trended lower than the percentage of fed mosquitoes treated with the healthy-associated combination (Figure 5D).

### 3.4 The effects of histamine and 5-HT on *A. stephensi* flight behavior may reflect an interaction of these biogenic amines

We previously reported that provisioning of malaria-associated histamine by priming and in blood increased flight activities of uninfected *A. stephensi* relative to controls in response to CO_2_ but did not increase investigation of visual objects or flight velocities (Rodriguez et al., 2021). In contrast, we observed that provisioning of 5-HT in blood had no effect on flight activities at either healthy- or malaria-associated levels, but flight velocities and object visitation were highest in mosquitoes provisioned with 0.15 µM 5-HT relative to 1.5 µM 5-HT or controls (Briggs et al., 2022).

**FIGURE 6:**
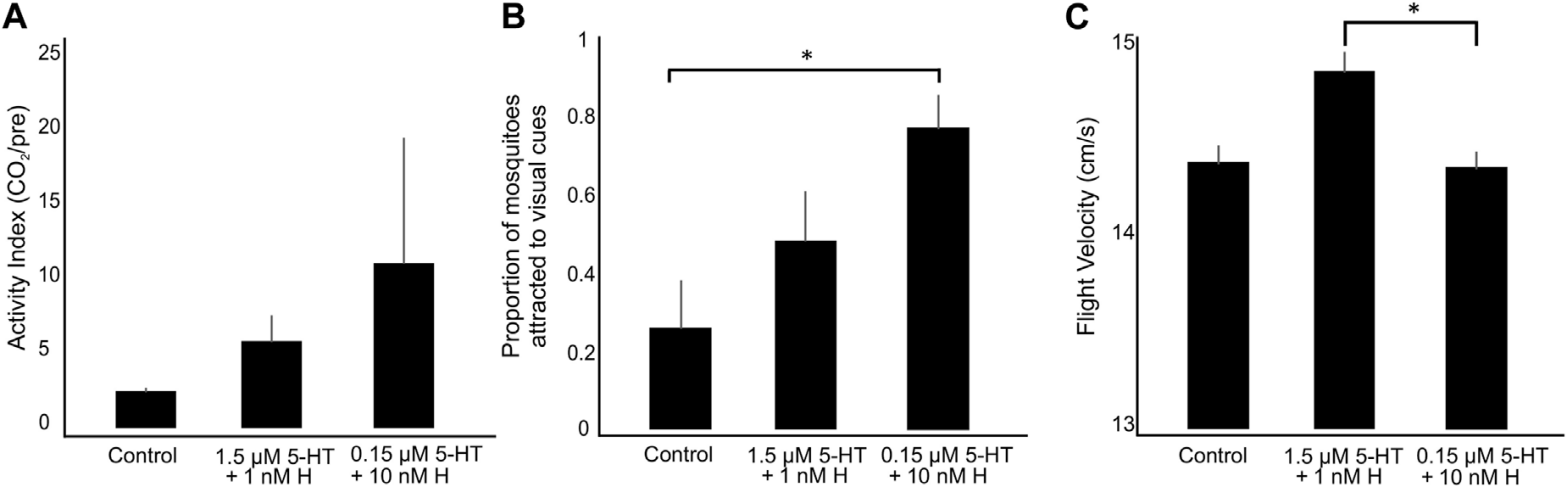
Flight behavior or *A. stephensi* provisioned with blood supplemented with combinations of histamine and 5-HT or with an equivalent volume of added water (control). **(A)** The activity index of mosquitoes in the arenas (N = 7 trials per treatment) calculated as the number of flying mosquitoes during CO_2_ divided by the number of flying mosquitoes pre-CO_2_ exposure. **(B)** The numbers of trajectories associated with mosquitoes investigating the visual cues. **(C)** Mosquito flight velocities before and during CO_2_ exposure. Bars are mean ± SEM. Asterisks denote a significant difference between groups (Dunn’s test: *p* < 0.05). H, histamine; 5-HT, serotonin.

To evaluate the combined effects of blood-derived histamine and 5-HT on flight, we tested *A. stephensi* behavior at 3 days following provisioning in 30 cm × 30 cm x 30 cm × 30 cm arenas that allowed fine-scale control of concentration and timing of olfactory stimuli. These arenas also allowed exploratory behavior, which has been demonstrated to provide neuromodulatory feedback during flight to brain regions associated with olfactory and visual responses (Van Breugel et al., 2015; Chapman et al., 2017; Vinauger et al., 2019; Wolff et al., 2023). During filtered air release, few mosquitoes flew in the arenas (across all replicates, mean = 133 ± 26 SEM) and there were no significant differences between treatments and controls that received no histamine and 5-HT (Kruskal-Wallis test, *p* > 0.37; Figure 6A). However, treatment with histamine and 5-HT more than doubled the numbers of flying mosquitoes (mean = 340 ± 38 SEM; Figure 6A), with the malaria-associated combination treatment trending towards higher activity compared to the healthy-associated combination treatment and controls (Figure 6A).

During CO_2_ exposure, the numbers of trajectories associated with mosquitoes visiting the white and black visual cues were significantly different across treatment groups (Figure 6B; Kruskal-Wallis test, *p* = 0.027). Mosquitoes provisioned with the healthy-associated combination of biogenic amines showed a tendency to inspect the visual objects more than control mosquitoes although this difference was not significant (Figure 6B; Dunn’s test, *p* > 0.1). Mosquitoes provisioned with the malaria-associated combination treatment, however, displayed a significantly higher tendency to inspect the visual objects than control mosquitoes (Figure 6B; Dunn’s test: *p* = 0.007).

As with the numbers of flying mosquitoes, flight velocities were significantly different across treatments (Figure 6C; Kruskal-Wallis test, *p* = 0.03). Although the flight velocities of mosquitoes provisioned with the malaria-associated combination treatment were not significantly different from the control (Dunn’s test, *p* > 0.9), mosquitoes provisioned with the healthy-associated treatment had significantly higher flight velocities than mosquitoes provisioned with the malaria-associated treatment (Dunn’s test, *p* = 0.025). Increased activity and inspection of visual objects during CO_2_ exposure following combination treatments, with trends toward higher activity in the malaria-associated combination treatment, are qualitatively similar to previous observations of increased activity with provisioning of malaria-associated histamine (Rodriguez et al., 2021). By contrast, we previously observed that flight velocities were not affected by malaria-associated histamine, but they were increased by malaria-associated 5-HT, which suggests that healthy levels of histamine and 5-HT may interact to increase flight velocity (Briggs et al., 2022).

### 3.5 Malaria-associated histamine and 5-HT additively increased *P. yoelii* infection in *A. stephensi*

We previously reported that priming with malaria-associated histamine increased the percentage of mosquitoes with at least one *P. yoelii* oocyst relative to both control and healthy-associated histamine treated mosquitoes, with increased mean midgut oocysts in both primed groups compared to controls (Rodriguez et al., 2021). Histamine priming had no effect on percentages of mosquitoes with salivary gland infections, but *P. yoelii* sporozoite density was higher in mosquitoes primed with malaria-associated histamine relative to controls (Rodriguez et al., 2021). In contrast, priming with healthy-associated 5-HT increased the percentage of mosquitoes with at least one *P. yoelii* oocyst relative to controls, with increased mean midgut oocysts in both groups of primed mosquitoes relative to control and a trend towards higher mean oocysts in the malaria-associated 5-HT treatment group (Briggs et al., 2022). In contrast to the effects of histamine, priming with 5-HT had no effect on salivary gland infections with *P. yoelii* sporozoites (Briggs et al., 2022). Effects of histamine provisioning on *P. falciparum* oocyst and sporozoite infection of *A. stephensi* were qualitatively similar to those of histamine priming on *P. yoelii* infection (Rodriguez et al., 2021). In contrast, the only effect of 5-HT provisioning on *P. falciparum* infection was a decrease in percentage of mosquitoes with at least one oocyst in the healthy-associated 5-HT treatment group relative to controls (Briggs et al., 2022). Histamine and 5-HT had no direct effects on parasite growth *in vitro*, so effects on infection were interpreted as indirect and due to biogenic amine-induced changes in the mosquito host (Rodriguez et al., 2021; Briggs et al., 2022). Taken together, malaria-associated histamine enhanced both *P. yoelii* and *P. falciparum* oocyst and sporozoite infection of *A. stephensi*, with the most epidemiologically relevant parameter of salivary gland infection unaffected by 5-HT for either parasite species. To understand the potential for interacting effects of histamine and 5-HT on sporogony, we primed mosquitoes with these biogenic amines alone and in combination prior to *P. yoelii* infection.

**FIGURE 7:**
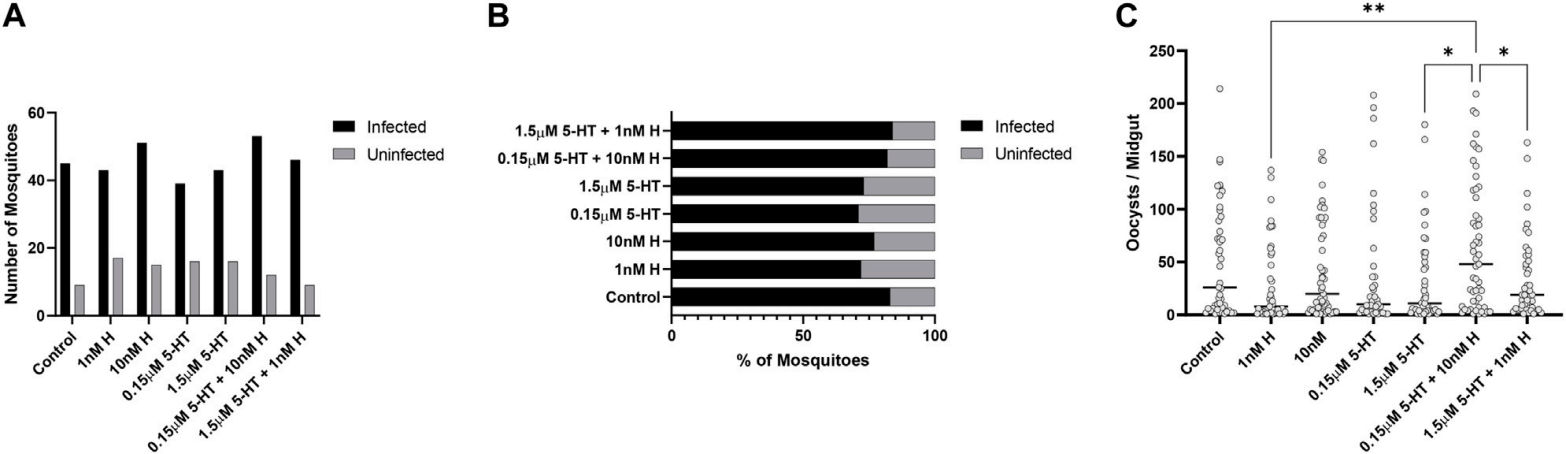
*Plasmodium y. yoelii* 17XNL oocyst infection in *A. stephensi* primed with histamine and 5-HT alone and in combination. **(A)** Numbers of *P. yoelii-*infected and uninfected mosquitoes in each treatment group, N = 4; Chi-square test (α = 0.05), no significance. **(B)** Percentages of infected and uninfected mosquitoes. **(C)** Numbers of *P. yoelii* midgut oocysts in each treatment group. N = 4; ordinary one-way ANOVA with Newman-Keuls multiple comparisons test (α = 0.05), **1 nM H vs. 0.15 μM 5-HT + 10nM H, *1.5 μM 5-HT vs. 0.15 μM 5-HT + 10nM H, *0.15 μM 5-HT + 10 nM H vs. 1.5 μM 5-HT + 1 nM H. H, histamine; 5-HT, serotonin.

**FIGURE 8:**
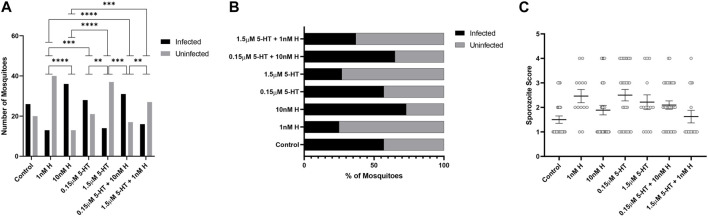
*Plasmodium y. yoelii* 17XNL sporozoite infection in *A. stephensi* primed with histamine and 5-HT alone and in combination. N = 4; Chi-square test (α = 0.05). **(A)** Numbers of *P. yoelii-*infected and uninfected mosquitoes in each treatment group, ****p < 0.0001, ***1 nM H vs. 0.15 μM 5-HT *p* = 0.0008, ***10 nM H vs. 1.5 μM 5-HT + 1 nM H *p* = 0.0005, **0.15 μM 5-HT vs. 1.5 μM 5-HT *p* = 0.0026, ***1.5 μM 5-HT vs. 0.15 μM 5-HT + 10 nM H *p* = 0.0002, **0.15 μM 5-HT + 10 nM H vs. 1.5 μM 5-HT + 1 nM H *p* = 0.0091. **(B)** Percentages of infected and uninfected mosquitoes. **(C)** Salivary gland sporozoite score in each treatment group. N = 4; ordinary one-way ANOVA with Newman-Keuls multiple comparisons test (α = 0.05), no significance. H, histamine; 5-HT, serotonin.

There were no treatment effects on the percentages of mosquitoes with *P. yoelii* oocysts (Figures 7A, B), but mosquitoes primed with the malaria-associated combination of histamine and 5-HT had higher mean numbers of oocysts than mosquitoes primed with healthy-associated histamine and 5-HT and the healthy-associated combination of these biogenic amines (Figure 7C). Treatment effects on salivary gland infection with *P. yoelii* sporozoites were more striking. Specifically, the malaria-associated histamine and 5-HT combination treatment was associated with higher percentages of infected mosquitoes relative to healthy-associated histamine and healthy-associated 5-HT, respectively (Figures 8A, B). Further, this pattern appeared to be driven by malaria-associated histamine (Figures 8A, B). In contrast to differences in percentages of infected mosquitoes, mean sporozoite infection scores in infected mosquitoes did not vary by treatment (Figure 8C).

### 3.6 Histamine and 5-HT appear to interact with *P. yoelii* infection to alter the tendency of *A. stephensi* to blood feed at 4 days and 11 days after infection

**FIGURE 9:**
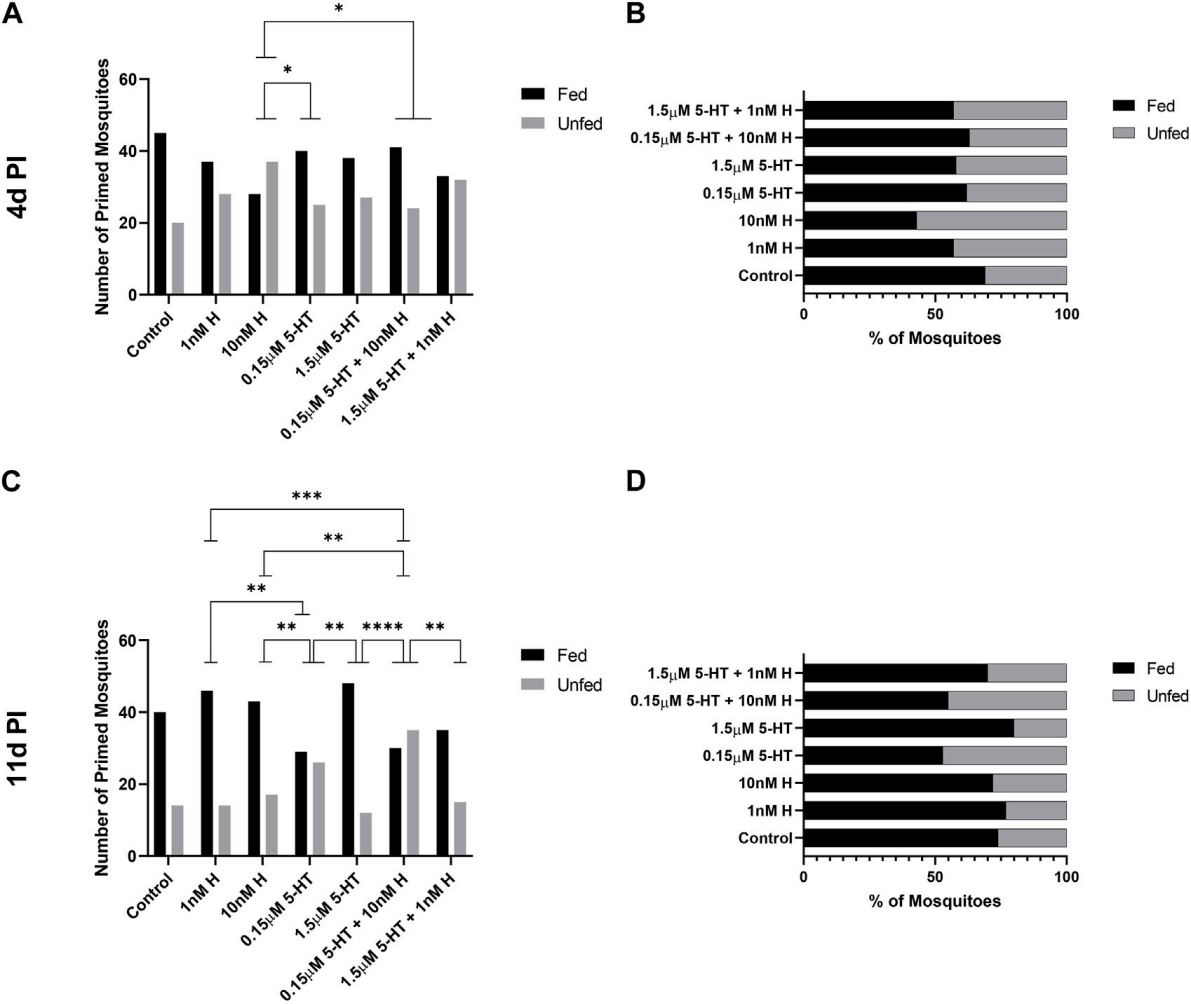
Tendency of *P. y. yoelii* 17XNL-infected *A. stephensi* primed with histamine and 5-HT alone and in combination to take a second bloodmeal at 4 days or 11 days post infection (PI). **(A)** Numbers of fed and unfed *P. yoelii-*infected mosquitoes at 4 days in each group. N = 4; Chi-square test, *10 nM H vs. 0.15 μM 5-HT *p* = 0.0351, *10 nM H vs. 0.15 μM 5-HT + 10 nM H *p* = 0.0223, **(B)** Percentages of fed and unfed mosquitoes at 4 days in each group. **(C)** Numbers of fed and unfed *P. yoelii-*infected mosquitoes at 11 days in each group. N = 4; Chi-square test. **1 nM H vs 0.15 μM 5-HT *p* = 0.0071, ***1 nM H vs. 0.15 μM 5-HT + 1 nM H *p* = 0.0005, *10 nM H vs. 0.15 μM 5-HT *p* = 0.0360, **10 nM H vs. 0.15 μM 5-HT + 10 nM H *p* = 0.0038, **0.15 μM 5-HT vs. 1.5 μM 5-HT *p* = 0.0019, ****1.5 μM 5-HT vs. 0.15 μM 5-HT + 10 nM H *p* = <0.0001, *0.15 μM 5-HT + 10 nM H vs. 1.5 μM 5-HT + 1 nM H *p* = 0.0106. **(D)** Percentages of fed and unfed mosquitoes at 11 days in each group. H, histamine; 5-HT, serotonin.

The tendency of uninfected *A. stephensi* provisioned by priming to take a second blood meal was enhanced by histamine, with histamine treatment associated with increased feeding tendency relative to treatment with 5-HT (Figures 4C, D). While the tendency of *P. yoelii*-infected *A. stephensi* to take a second blood meal at 4 days later was modestly altered by treatment (Figures 9A, B), these effects contrasted with those in uninfected *A. stephensi*. Specifically, the tendency of infected *A. stephensi* to re-feed at 4 days was enhanced by malaria-associated 5-HT relative to malaria-associated histamine (10 nM histamine vs. 0.15 µM 5-HT, *p* < 0.05) or by the addition of malaria-associated 5-HT to histamine (10 nM histamine vs. 0.15 µM 5-HT + 10 nM histamine, *p* < 0.05; Figures 9A, B).

At 14 days, the tendency of uninfected *A. stephensi* to take a second blood meal trended lower for mosquitoes primed with malaria-associated treatments relative to females primed with healthy-associated treatments (Figures 5C, D). Similarly, the tendency of infected *A. stephensi* to take a second blood meal at 11 days was lower for mosquitoes primed with malaria-associated treatments relative to females primed with healthy-associated treatments (Figures 9C, D). With respect to individual treatments, the tendency of infected *A. stephensi* to take a second blood meal at 11 days was higher following histamine treatment alone at either dose relative to malaria-associated 5-HT or the malaria-associated combination (10 nM histamine vs. 0.15 µM 5-HT + 10 nM histamine, 10 nM histamine vs. 0.15 µM 5-HT, 1 nM histamine vs. 0.15 µM 5-HT, 1 nM histamine vs. 0.15 µM 5-HT + 10 nM histamine, *p* < 0.05; Figures 9C, D). At the same time, treatments with healthy 5-HT alone was associated with an increased percentage of fed, infected mosquitoes relative to those treated with malaria-associated 5-HT (1.5 µM 5-HT vs. 0.15 µM 5-HTp < 0.05; Figures 9C, D). In sum, priming with low 5-HT, alone or in combination with histamine, was associated with lower percentages of fed, infected mosquitoes at 11 days relative to other treatment groups. Taken together, feeding patterns of primed uninfected and primed *P. yoelii*-infected *A. stephensi*, which also exhibited large differences in salivary gland infection by treatment (Figures 8A, B), suggested an interaction between treatment and infection on the tendency to re-feed at 11 days post-infection.

### 3.7 Biogenic amines in *A. stephensi* may have concentration-dependent effects on timing, duration, and peak size of malaria outbreaks, but long-term human infection prevalence may not be impacted substantially

**FIGURE 10:**
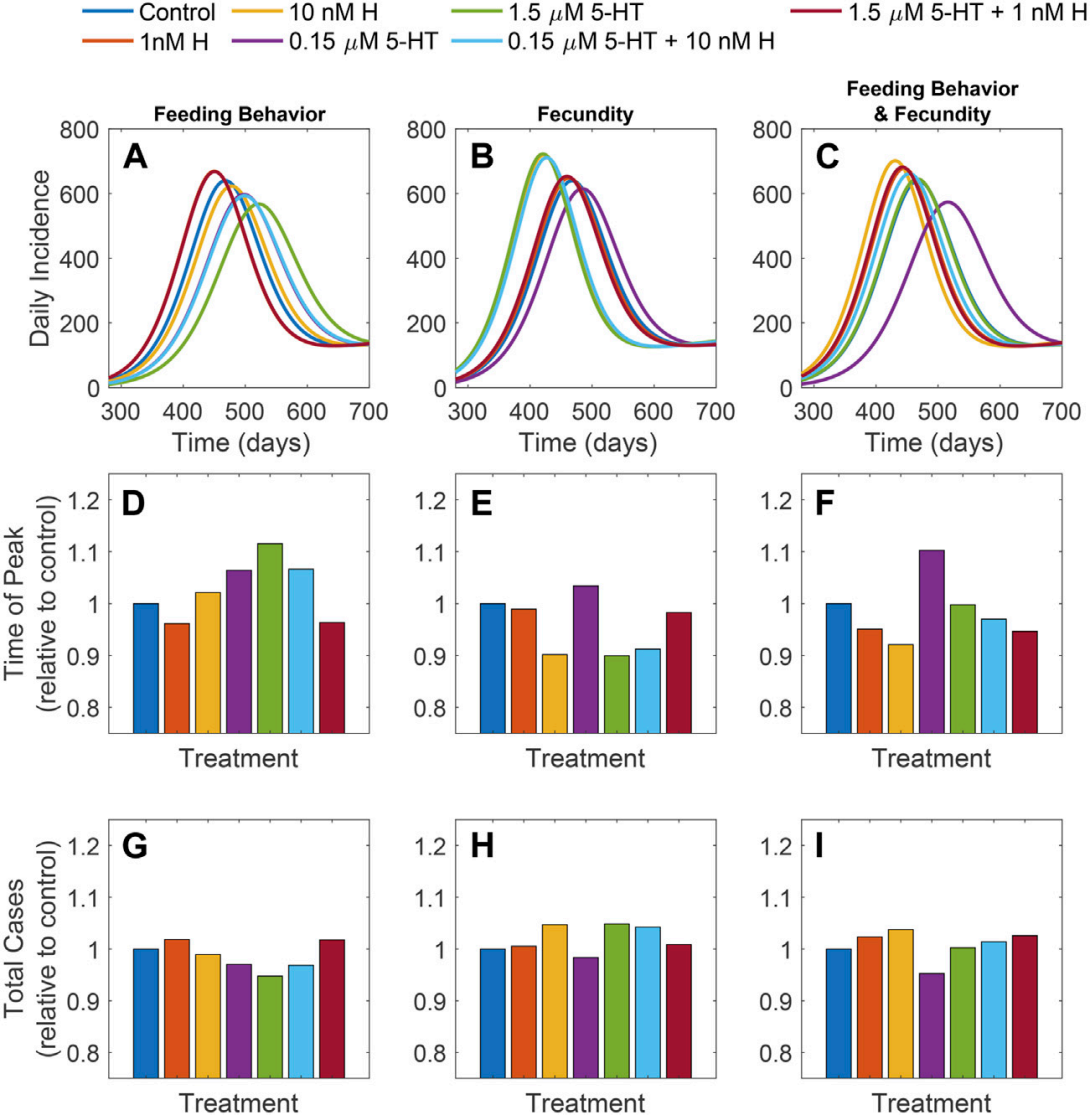
Results of model simulations. Daily incidence **(A–C)**, time of the peak daily incidence relative to the control **(D–F)**, and total cases relative to the control **(G–I)** for each treatment. Effects of amines observed in experiments were considered in three scenarios: differences in fecundity **(A,D,G)**, differences in tendency to take bloodmeal at 4 and 14 days **(B,E,H)**, and both changes in fecundity and tendency to take a bloodmeal at 4 and 14 days **(C,F,I)**.

Results in Figure 10; Supplementary Figures S7, S8 show modeling projections of the effects of feeding behavior, fecundity or these two parameters on malaria outbreak parameters. Timing and duration of outbreaks and peak numbers of human cases occurring during outbreaks were impacted by biogenic amines in *A. stephensi*; each of these short-term outcomes differed across mosquito groups in the present study. However, endemic prevalence (as well as total number of human cases) did not differ substantially across treatments (Figures 10G–I). All three predicted measures of outbreak dynamics were similar or nearly identical for both the low histamine and the healthy combination mosquito groups (also with low histamine), suggesting that the presence of mosquitoes with healthy levels of histamine does not lead to substantial changes in outbreak parameters.

Interestingly, the impacts on outbreak dynamics in human populations differed when considering mosquito fecundity *versus* the tendency to take a second blood meal. When differences in the tendency to take a second blood meal alone were considered, the projected peaks in daily incidence of human infections for the healthy combination and low histamine mosquito groups were earlier and higher than for all other mosquito groups (Figures 10A, D). In the presence of mosquitoes with malaria-associated levels of both biogenic amines or low 5-HT alone, human cases peaked later and lower than cases associated with other groups except high 5-HT alone. This suggests that for blood feeding behavior, the presence of malaria-associated levels of histamine in *A. stephensi* at the initiation of an outbreak does not lead to substantial differences in human infection dynamics from that associated with *A. stephensi* with malaria-associated levels of 5-HT.

When differences in fecundity associated with biogenic amine levels were considered alone, the differences in outbreak timing and peak size across mosquito groups were greater than was observed when considering the tendency to take a second blood meal alone. The differences in fecundity observed in the high 5-HT, high histamine, and malaria-associated combination groups seemed to lead to higher and faster peaks in human malaria incidence relative to the other treatments (Figures 10B, E), and these three groups were also associated with the greatest outbreak size (Figure 10H). Outbreak parameters associated with mosquitoes with the healthy combination or low histamine alone were similar to the control group, whereas outbreak parameters associated with mosquitoes with low 5-HT alone were lower and peaked later than with all other mosquito groups.

When considering the combined impacts of both fecundity and tendency to take a second blood meal, the time to human outbreak peak was highest and earliest with the high histamine mosquito group, suggesting that the impact on fecundity in the high histamine group drives outbreak dynamics with the malaria-associated combination mosquito group (Figures 10D, F). Time to outbreak peaks associated with the low histamine and the healthy combination mosquito groups followed in size and timing, but were earlier and higher than were observed for all other groups in this scenario. Interestingly, human outbreak dynamics associated with the high 5-HT alone mosquito group were almost identical to control, suggesting that the influence of 5-HT on feeding behavior substantially tempers the impacts on fecundity that were observed (Figures 10D–F). Across all mosquito groups, the outbreak peak associated with the low 5-HT group was the latest and lowest, emphasizing that the impacts on fecundity of high histamine greatly influence malaria outbreak dynamics.

Although differences were observed across mosquito groups for all three scenarios for human outbreak dynamics, the long-term prevalence of human infection was not substantially affected by biogenic amine-associated differences in mosquito fecundity or tendency to take a second blood meal (Supplementary Figures 7G–I).

## 4 Discussion

Ingestion of histamine and 5-HT alone and in combination by female *A. stephensi* impacts important aspects of their behavior and physiology. The recorded changes are biologically relevant in that these effects are mediated at healthy- and malaria-associated blood levels of these biogenic amines, concentrations that would be ingested by mosquitoes during feeding. We compared impacts of histamine and 5-HT on fecundity, lifespan, feeding behavior, flight behavior, and *P. yoelii* infection of female *A. stephensi*. While not all of these parameters were impacted to the same degree, the sum of these effects suggests that ingested histamine and 5-HT alter the likelihood of transmission by mosquitoes that feed on hosts with symptomatic malaria *versus* a healthy host.

In the fecundity studies, we observed effects on clutch size and female oviposition that were associated with interactions between histamine and 5-HT, patterns supported by previous observations that 5-HT alone had no effect on female oviposition and clutch size (Briggs et al., 2022). Hersey et al. (2021) reported histamine binding to 5-HT neuron receptors in the human brain, which inhibited the release of 5-HT. Accordingly, histaminergic and serotonergic signaling may interact in *A. stephensi* to control female reproduction.

Neither combination of histamine and 5-HT altered lifespan or lifetime weekly blood feeding, but as previously observed (Rodriguez et al., 2021; Briggs et al., 2022), treatment differences in the tendency to take a second blood meal at 4 days and 11 or 14 days were not captured in these lifespan studies. These days were selected based on timing of parasite development, such that 4 days PI would be associated with oocyst infection, while 11 days and 14 days PI would be associated with sporozoite infection of the salivary glands. Taking a second blood meal is necessary for transmission, but there are risks at these time points, particularly at 4 days where death of the mosquito due to host defensive behaviors during mosquito feeding would result in a loss of those parasites from the transmission cycle. With uninfected mosquitoes, an increased tendency to feed at either 4 days or at 11 or 14 days after an uninfected blood meal would increase the chances that this mosquito encounters an infected host, behavior that would also increase transmission.

At 4 days, there were more treatment effects on the tendency of uninfected *A. stephensi* to take a second blood meal compared to infected *A. stephensi*. Further, this behavior at 4 days was enhanced by histamine in uninfected mosquitoes and by 5-HT in infected mosquitoes, with the malaria-associated combination treatment trending as the highest percentage of fed, infected mosquitoes among all of the groups. At 14 days, the percentages of fed, uninfected mosquitoes were lower for malaria-associated *versus* healthy-associated treatments, while the percentages of fed, infected mosquitoes were lower when treatment included the malaria-associated concentration of 5-HT. In the context of infection, these behaviors could be interpreted as adaptive for parasite survival. Specifically, at 4 days, fewer stimuli that enhance feeding of infected *A. stephensi* would be associated with reduced risk to developing oocysts, while at 14 days, the opposite would be true in that sporozoite transmission would be increased by an increased tendency to feed. However, the nearly 2-fold higher percentage of *A. stephensi* with salivary gland sporozoites following priming with the malaria-associated combination compared to the healthy-associated combination treatment (Figure 8B) contrasts with the percentages of infected mosquitoes in these groups that took a second blood meal at 11 days (Figure 9B). If we discount an effect of sporozoite load on feeding behavior (Figure 8C), the patterns of feeding at 11 days beg the question as to whether equal proportions of infected and uninfected mosquitoes re-fed in each treatment group. We will address this complexity in future studies and with more comprehensive modeling of these parameters.

In our studies of flight behavior, the blood meal provisioned with the malaria-associated combination treatment evoked changes in *A. stephensi* responses to CO_2_ and increased their responses to visual cues. These patterns were analogous to those in our previous studies of malaria-associated histamine (10 nM) (Rodriguez et al., 2021) and malaria-associated 5-HT (0.15 μM) (Briggs et al., 2022), which included changes in the numbers of activated mosquitoes (10 nM histamine), the numbers of mosquitoes investigating attractive visual objects (0.15 μM 5-HT), and their flight velocity (0.15 μM 5-HT) (Supplementary Figure S3A). Activity-dependent neuromodulation plays an important role in sensory-guided behaviors. For example, during flight, sensorimotor feedback to the brain facilitates the processing of olfactory and visual information, enabling faster response times to image speeds and turbulent odors that are necessary when the mosquito is in flight (Tuthill et al., 2014; Van Breugel et al., 2015; Vinauger et al., 2019). The tuning of these connections between the brain and gut or motor systems may be altered due to physiology of the insect. In the blow fly and *Aedes aegypti*, the ventral nerve cord and recurrent feedback from the gut-to-brain system is critical to modulate consumption, and without this feedback, the insect will continue feeding and host-seeking (Bodenstein and Dethier, 1958; Klowden and Lea, 1979). These connections are modulated by the release of biogenic amines, including 5-HT, which can impact feeding responses and muscle contractions in the gut (French et al., 2014). In the kissing bugs *Rhodnius prolixus* and *Triatoma infestans*, serotonergic neurons innervate the thoracic ganglion and digestive tract, and are thought to modulate diverse physiological processes through release of 5-HT into the hemolymph during a blood meal. For kissing bugs, release of 5-HT is also thought to facilitate the digestion of blood and rapid diuresis, and possibly sensitization of the visual system (Lange et al., 1988; Reisenman et al., 2002; Orchard, 2006). In our previous studies (Rodriguez et al., 2021; Briggs et al., 2022), we identified histaminergic and serotonergic neurons in the *A. stephensi* thoracic ganglion and strong labeling in the gut. In the current study, provisioning of histamine and 5-HT in blood, with activity-dependent flight behavior, may facilitate responses to host-associated cues, like CO_2_ or visual cues, providing a direct link between the gut-brain axis. Although priming over the course of the blood meal and subsequent days may enhance the links between the brain and gut (Figures 4C, D), a single blood meal and activity associated with flight may enhance the long-term impacts of ingested biogenic amines.

There are different ascending pathways from the gut to brain that can, in turn, modulate sensory neurons in the brain where 5-HT and histamine play important roles. In the current study, concentrations of histamine and 5-HT associated with healthy human hosts had different impacts on flight behaviors than did concentrations of these biogenic amines associated with human hosts suffering from severe malaria. The physiological and biochemical mechanisms by which the gut can modulate the brain are diverse (Supplementary Figure S3B). For example, different ascending neurons in the brain release diverse neurohormones, including neuropeptides (short neuropeptide F), insulin-like peptides, and diuretic hormones (Cui et al., 2022). In *Drosophila melanogaster*, these neurons can respond to stimulation by nutrients (sugars and amino acids), and behaviorally increase attraction to food odors by increasing the responses of olfactory sensory neurons. The location of these neurons in the brain corresponds with innervation by serotonergic neurons that also project to different brain regions, including olfactory loci (Luo et al., 2012). In *A. stephensi*, we found that serotonergic neurons are present in the optic lobe (Briggs et al., 2022), which also generates histamine (Rodriguez et al., 2021). Histamine receptors are critical for visual processing (Schnaitmann et al., 2018). Thus, the connections between gut and brain may be diverse, but 5-HT and histamine are critically involved in both the gut and brain to influence behavior.

Our modeling projected human malaria outbreak parameters based on patterns of mosquito fecundity and blood feeding following priming with 5-HT and histamine (Figure 10). The observation that histamine priming led to increased feeding tendency at 4 days compared to priming with 5-HT (Figures 4C, D) corresponded with higher and earlier peaks of daily human malaria incidence. Further, larger outbreaks were associated with the low histamine mosquito group, when considering histamine *versus* 5-HT groups (Figures 10A, D, G). The effects at 14 days of mosquito priming (Figures 5C, D) on daily human malaria incidence were less obvious, perhaps due to differences in mosquito survival at this timepoint. The effect of fewer mosquitoes feeding following treatment with the malaria-associated combination compared to the healthy combination (Figure 5D) was evident in daily human malaria incidence, where more cases and an earlier peak were associated with the healthy combination mosquito group relative to the malaria combination mosquito group (Figures 10A, D, G). Our modeling of the impacts of biogenic amines on mosquito fecundity alone was somewhat surprising because treatments that resulted in fewer eggs laid (high histamine, high 5-HT, malaria-associated combination, Figure 1) were associated with higher and earlier outbreaks (Figures 10B, E, H). A possible driving force behind this result, however, is that in populations experiencing strong density-dependent regulation, higher number of eggs and thus larvae can result in higher larval mortality (or slower development), which in turn results in lower adult female mosquito population sizes (Muriu et al., 2013). Although *Anopheles* spp. populations are generally density-regulated, it could be worth revisiting this result with a model that does not include density dependence to better understand the impacts of increased fecundity on populations without density-dependent regulation.

Our models of the effects of mosquito feeding behavior and fecundity on human malaria outbreak parameters emphasize the importance of analyzing mosquito life history traits and behavioral outputs both individually and in combination. Specifically, separate analyses of feeding behavior and fecundity revealed distinct human malaria incidence patterns across mosquito groups, while analyses of the combined effects of feeding behavior and fecundity seemed to counterbalance. For example, models that included both fecundity and feeding behavior in mosquitoes treated with the malaria-associated combination projected a relatively later and lower malaria outbreak peak compared to modeling of feeding behavior alone, but a higher and earlier peak when modeling fecundity alone. In contrast, daily human malaria incidence associated with mosquitoes provisioned with the malaria combination was centered among all the treatments (Figure 10). It is also possible that some effects of biogenic amines on mosquitoes may be more impactful than others on human malaria. For example, modeling of feeding behavior alone revealed that peak malaria incidence associated with the high 5-HT mosquito group was later and lower than that associated with any other mosquito group, whereas for analysis of fecundity alone, the same mosquito group was associated with one of the highest and earliest peaks. Modeling of both fecundity and feeding behavior, however, projected daily incidence that was almost identical to the control, which had a lower and later peak relative to most mosquito groups except low 5-HT mosquitoes, suggesting that the impacts of feeding behavior negated those of fecundity (Figure 10).

Taken together, our data demonstrate that histamine and 5-HT have a wide range of effects on mosquito behavior and life history and thereby transmission dynamics in human populations. Continued study of the interactions between subtle changes to mosquito behavior and physiology and malaria transmission at the populations level will add detail and precision to projections of disease dynamics in host populations.

## Data Availability

The raw data supporting the conclusion of this article will be made available by the authors, without undue reservation.
