# Supplementary material for: Ingested histamine and serotonin interact to alter Anopheles stephensi feeding and flight behavior and infection with Plasmodium parasites
Source: Front Physiol. 2023 Jul 24;14:1247316. doi: 10.3389/fphys.2023.1247316 (PMC10405175; doi:10.3389/fphys.2023.1247316)
Supplement: Supplementary file 1 [file DataSheet1.DOCX]

| **Treatments** | **GC1** | **GC2** | **GC3** |
| --- | --- | --- | --- |
| **Control** | 40 ± 1.49 def* | 39.75 ± 1.88 ef | 46.47 ± 2.30 bcd |
| **1nM H** | 38.99 ± 1.48 ef | 48.54 ± 2.23 ab | 53.47 ± 2.50 a |
| **10nM H** | 39.01 ± 1.82 ef | 42.33 ± 1.87 cdef | 46.24 ± 2.67 bcd |
| **0.15μM 5-HT** | 39.94 ± 1.34 ef | 48.57 ± 2.00 ab | 46.48 ± 2.34 bc |
| **1.5μM 5-HT** | 39.59 ± 1.86 ef | 44.53 ± 2.43 bcde | 44.13 ± 2.94 bcdef |
| **0.15μM 5-HT + 10nM H** | 39.11 ± 1.70 ef | 46.66 ± 1.92 bc | 46.33 ± 2.44 bc |
| **1.5μM 5-HT + 1nM H** | 39.94 ± 1.80 ef | 49.64 ± 1.70 ab | 46.38 ± 1.74 bc |

**Supplementary Table 1.** Mean clutch size ± standard error for each gonotrophic cycle (GC) by treatment with histamine (H), serotonin (5-HT) or combinations of these biogenic amines.

*Significance was determined at α = 0.05. Values sharing any of the same following lowercase letters are not significantly different.

**Supplementary Table 2.** Survival analysis of uninfected *A. stephensi* provisioned a weekly blood meal supplemented with the malaria-associated combination of biogenic amines 0.15μM 5-HT + 10nM histamine (H), the healthy-associated combination 1.5μM 5-HT + 1nM H or an equivalent volume of water added as a control.

|  | **Comparison** | **Log-rank (Mantel-Cox)** | **Significant*** | **Gehan-Breslow-Wilcoxon** | **Significant** |
| --- | --- | --- | --- | --- | --- |
| **Rep 1** | Control vs 0.15μM 5-HT + 10nM H | 0.0036 | Y | 0.0018 | Y |
|  | Control vs 1.5μM 5-HT + 1nM H | 0.483 | N | 0.645 | N |
|  | 0.15μM 5-HT + 10nM H vs 1.5μM 5-HT + 1nM H | 0.0562 | N | 0.0152 | Y |
| **Rep 2** | Control vs 0.15μM 5-HT + 10nM H | 0.449 | N | 0.4688 | N |
|  | Control vs 1.5μM 5-HT + 1nM H | 0.5131 | N | 0.3647 | N |
|  | 0.15μM 5-HT + 10nM H vs 1.5μM 5-HT + 1nM H | 0.1509 | N | 0.185 | N |
| **Rep 3** | Control vs 0.15μM 5-HT + 10nM H | >0.9999 | N | >0.9999 | N |
|  | Control vs 1.5μM 5-HT + 1nM H | 0.4005 | N | 0.7783 | N |
|  | 0.15μM 5-HT + 10nM H vs 1.5μM 5-HT + 1nM H | 0.1334 | N | 0.1492 | N |

*Y = yes, N = no.


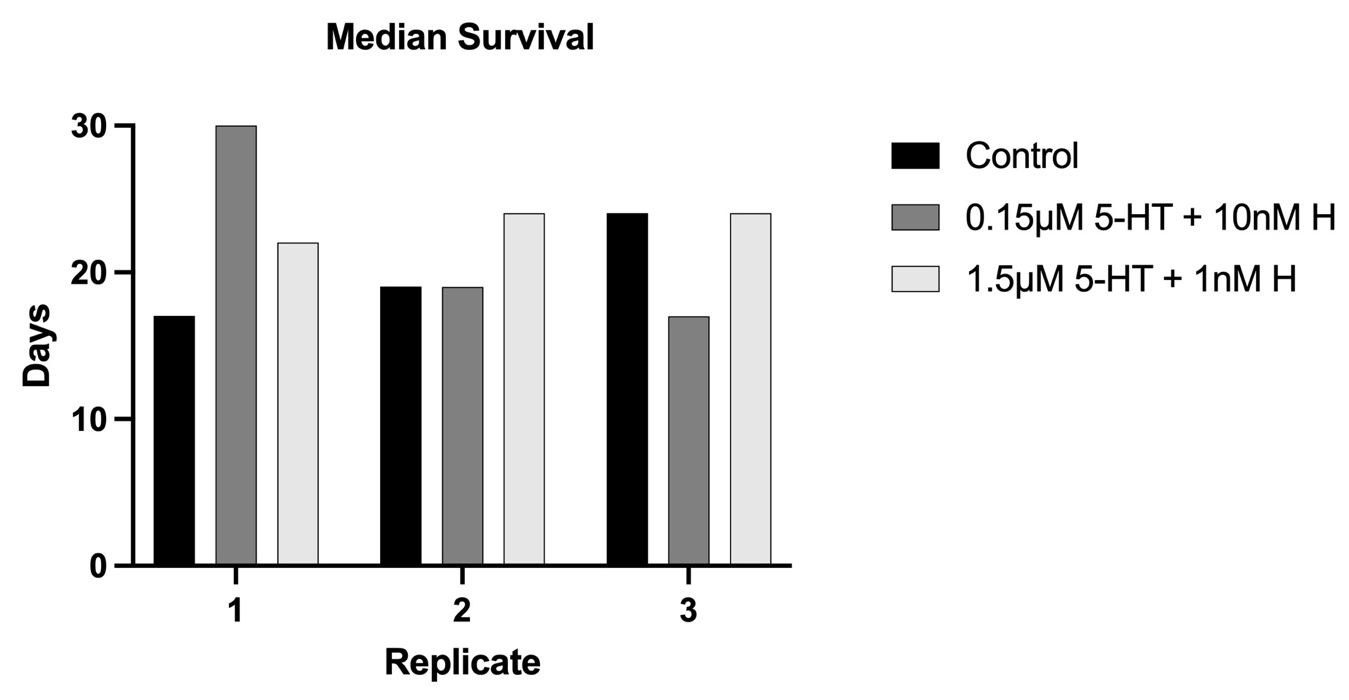


**Supplementary Figure 1. Median day of survival of uninfected *A. stephensi*.** Mosquitoes were given a weekly blood meal over lifespan supplemented with the malaria-associated combination 0.15μM 5-HT + 10nM histamine (H), the healthy-associated combination 1.5μM 5-HT + 1nM H or an equivalent volume of water added as a control. N = 3, Kruskal-Wallis test with Dunn’s multiple comparisons test. No significant differences.

**Supplementary Table 3.** Feeding behavior of uninfected mosquitoes provisioned a weekly blood meal supplemented with the malaria-associated combination of biogenic amines 0.15μM 5-HT + 10nM histamine (H), the healthy-associated combination 1.5μM 5-HT + 1nM H or an equivalent volume of water added as a control.

|  | **Comparison** | **Log-rank (Mantel-Cox)** | **Significant*** | **Gehan-Breslow-Wilcoxon** | **Significant** |
| --- | --- | --- | --- | --- | --- |
| **Rep 1** | Control vs 0.15μM 5-HT + 10nM H | 0.404 | N | 0.7953 | N |
|  | Control vs 1.5μM 5-HT + 1nM H | 0.3123 | N | 0.9569 | N |
|  | 0.15μM 5-HT + 10nM H vs 1.5μM 5-HT + 1nM H | 0.729 | N | 0.8397 | N |
| **Rep 2** | Control vs 0.15μM 5-HT + 10nM H | 0.0838 | N | 0.0337 | Y |
|  | Control vs 1.5μM 5-HT + 1nM H | 0.0029 | Y | 0.0017 | Y |
|  | 0.15μM 5-HT + 10nM H vs 1.5μM 5-HT + 1nM H | 0.3003 | N | 0.5164 | N |
| **Rep 3** | Control vs 0.15μM 5-HT + 10nM H | 0.0472 | Y | 0.0063 | Y |
|  | Control vs 1.5μM 5-HT + 1nM H | 0.9188 | N | 0.3948 | N |
|  | 0.15μM 5-HT + 10nM H vs 1.5μM 5-HT + 1nM H | 0.0445 | Y | 0.0443 | Y |

*Y = yes, N = no.


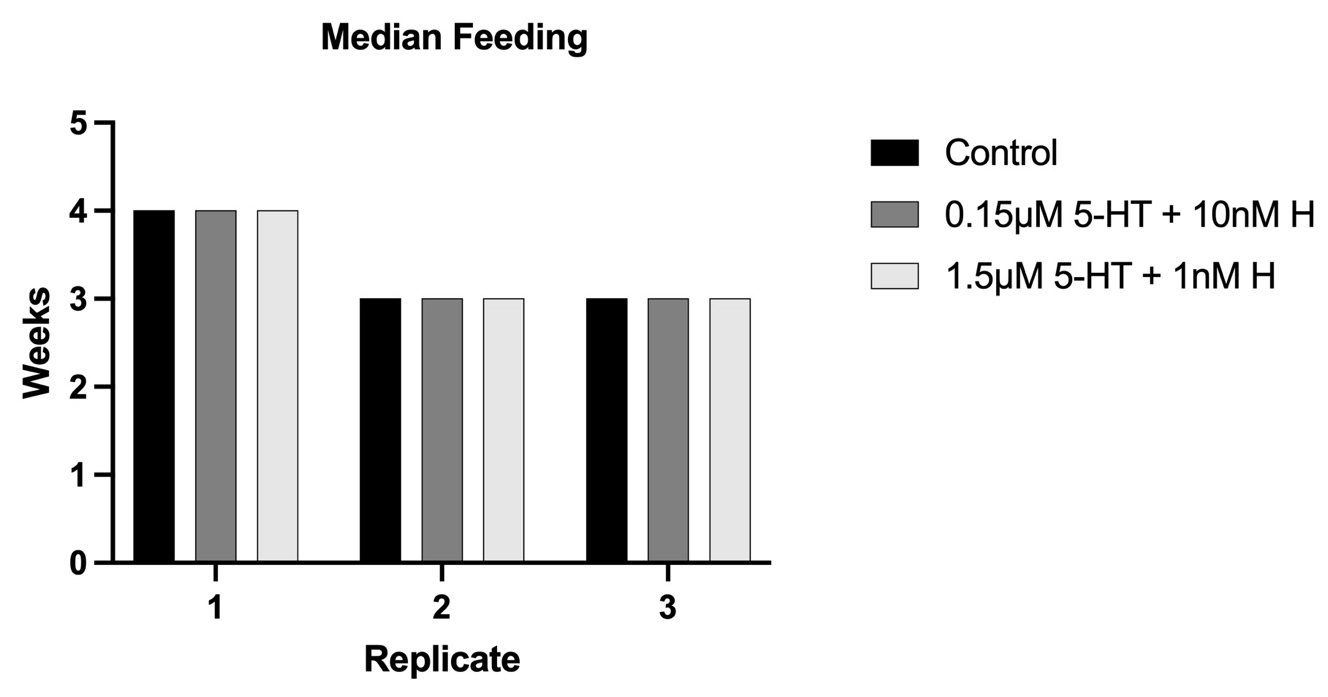


**Supplementary Figure 2. Median week of blood feeding cessation of uninfected *A. stephensi*.** Mosquitoes were provisioned over their lifespan a weekly blood meal supplemented with the malaria-associated combination of biogenic amines 0.15μM 5-HT + 10nM histamine (H), the healthy-associated combination 1.5μM 5-HT + 1nM H or an equivalent volume of water added as a control. N = 3, Kruskal-Wallis test with Dunn’s multiple comparisons test. No significant differences.


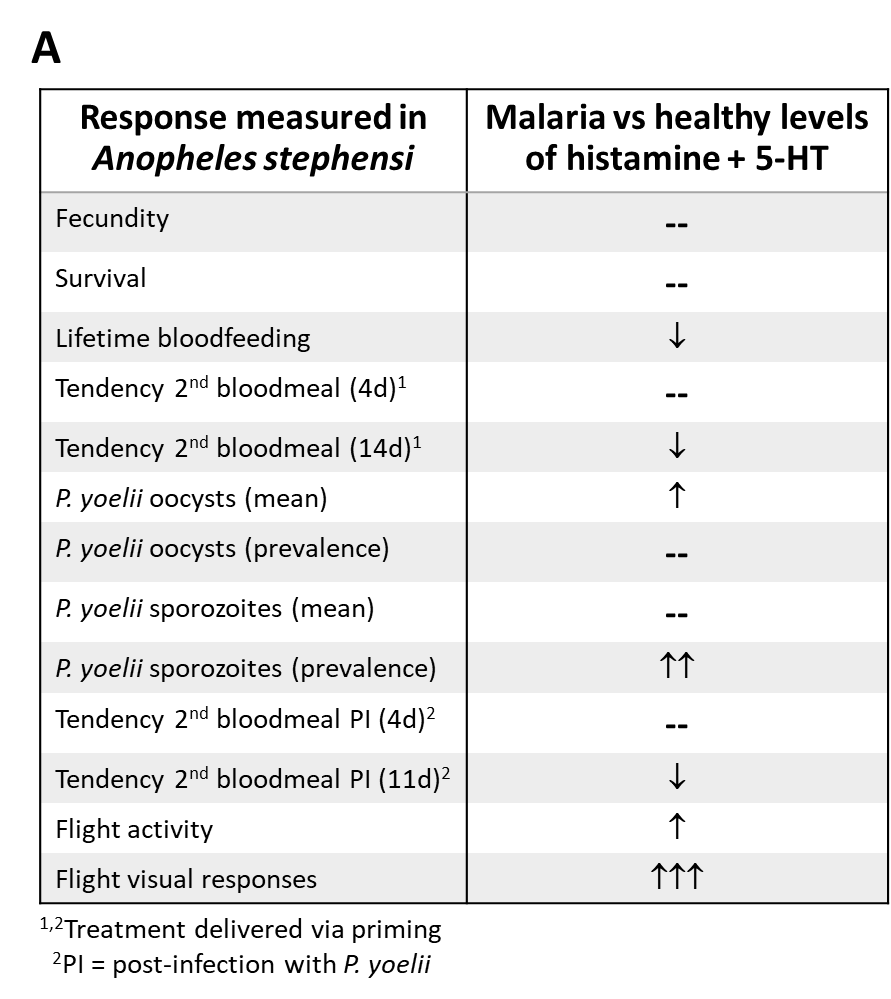

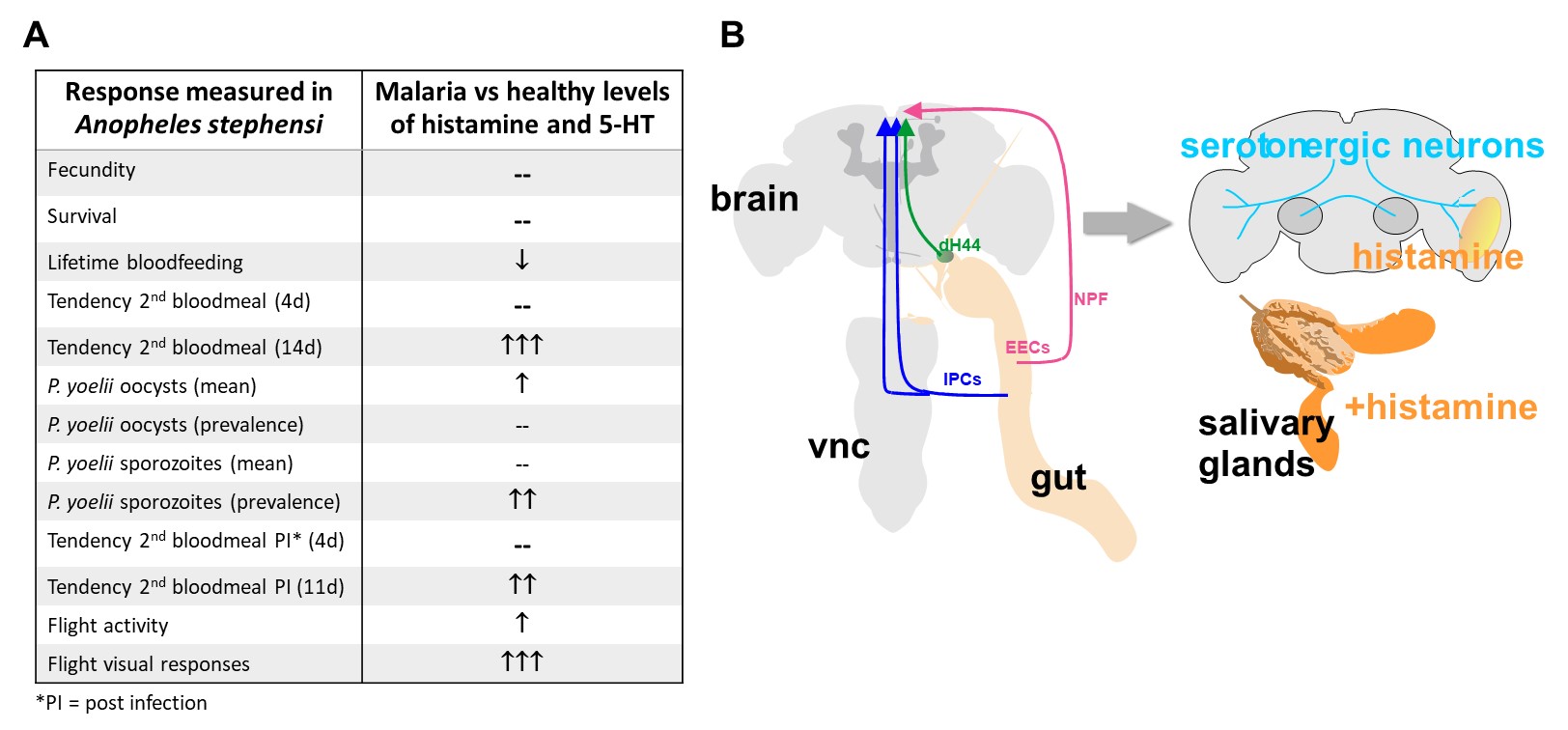


**Supplementary Figure 3. Summary of effects of the ingestion of histamine and 5-HT in combination on tested processes, and serotonergic and histaminergic links between gut and brain.** (A) Summary table comparing the effects of ingestion of healthy- and malaria-associated levels of 5-HT and histamine on *A. stephensi* behavior and infection. (B) Feedback from the gut to the brain is mediated through diverse pathways, including insulin producing cells (IPCs, blue), enteroendocrine cells (EECs, pink) that release neuropeptide F (NPF), and putative cells that release diuretic hormones (dH44 cells, green). These cells project to brain areas that are innervated by serotonergic neurons that also project to visual and olfactory areas that express histamine. The salivary glands also express histamine.

**Mathematical model**

We developed a discrete time host-vector epidemiological model in which humans may be classified as Susceptible, Exposed, Infectious, or Recovered. Mosquitoes may be classified as Susceptible, Exposed, or Infectious. For both humans and mosquitoes, an individual in the susceptible (*S_h_* and *S_v_*) class is uninfected and can become infected. In the exposed class (*E_h_* and *E_v_*), an individual has been infected but is not infectious. This is the class in which the pathogen undergoes an incubation period in the individual. In the infectious class (*I_h_* and *I_v_*), the individual can now transmit the pathogen. In the recovered class (*R_h_*), humans are no longer infectious.


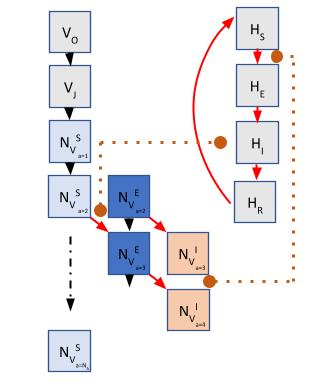


**Supplementary Figure 4.** Schematic representation of the model.

**Infection Dynamics.** Each day, the probability that susceptible humans become infected is given by **λ_VH_(t) = 1-exp(-β_VH_ c (Σ_a_p_c_(a)I_v_(a,t))/N_H_(t))**, where *β_VH_* is the probability that pathogen transmission from vector to host occurs upon a bite, *c* is the daily biting rate of an adult female, *I_v_*(*a,t*) is the total number of infectious (and blood-feeding) vectors of age *a* on day *t*, p_c_(a) is the fraction of females of age *a* taking bloodmeals, and *N_H_(t)* is the total number of humans on day *t*. Once infected, the probability that a human becomes infectious (i.e., completes the incubation period) is given by 𝜎_H_. Once infectious, humans can infect mosquitoes. The daily probability that a human clears an infection is given by γ_H_. We assume a period of temporary immunity, and the daily probability that a human loses immunity is given by ψ_H_.

Infection dynamics of the vector population are similar. The daily probability that a vector becomes infected is given by **λ_HV_(t) = 1-exp(-β_HV_ c I_H_(t)/N_H_(t))**, where *β_HV_* is the probability that pathogen transmission from human to mosquito host occurs upon a bite and *I_H_(t)* is the total number of infectious humans on day *t*. All other parameters and variables are defined as in **λ_VH_(t)**. Once infected, the extrinsic incubation period begins, and the probability that a mosquito becomes infectious is given by 𝜎_V_. Once infectious, a mosquito remains infectious for life.

**Population Dynamics.** Human population dynamics are not complex in this model. We assume no births or deaths in the human population and that **N_H_ = S_H_ + E_H_ +I_H_ + R_H_**. That is, the population remains constant.

To incorporate different life stages in the mosquito class, we structure the population by age. There are two classes prior to emergence: Eggs (*V_O_*) and Juveniles (*V_j_*). After emergence as adults, the mosquitoes progress through a new age class each day (*A_v_^i^,* where *i=1,2,...,N*) and *A* can be *S*, *E*, or *I*, depending on infection status.

The number of eggs laid by an adult female of age *a* each day is **ɑ(a) = f(a)p_f_(a)**, where **f(a)** is the number of eggs laid by a female of age **a**, and the **p_f_a(a)** is the fraction of females of age **a** that are laying eggs. The values for these are taken from data collected in the present work, and Figure 2 (b,c) shows the values we use for this study.


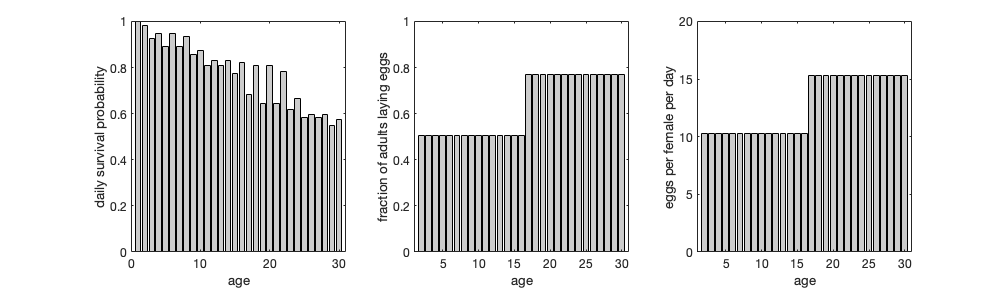


**Supplementary Figure 5.** Life history characteristics as a function of age that are used in the model. Left panel, daily survival probability **s(a)**; center panel, fraction of adult females that lay eggs, **p_f_a(a)**; and, right panel, the number of eggs laid per female per day, **f(a)**.

Eggs either die or hatch each day, with probability (1-*μ_O_*) or *ν_O_*, respectively. There is one class of juvenile mosquitoes for all larval and pupal stages. Juvenile mosquitoes either emerge as adults or die, with probabilities *ν_J_* or **(1-μ_J_exp( (dV_J_(t))^b^))**, respectively. In the mortality probability, *μ_J_* is the density-independent daily survival probability, and **exp((dV_J_(t))^b^)** is the density-dependent daily survival probability with parameters *d* and *b* that, together with other demographic parameters, determine the population size. Upon emergence, one-half of all emerging adults are female. All adult females die with probability **(1-s(a))**, where **s(a)** is the daily survival probability shown in **Fig. 2A**.

Taken together, we have the following mathematical model

$$H_{S}(t+1) =H_{S}(t)(1-\lambda_{VH}(t)) + \psi_{H}H_{R}(t)$$

$$H_{E}(t+1) =H_{E}(t)(1-\sigma_{H}) + \lambda_{VH}(t)H_{S}(t)$$

$$H_{I}(t+1) =H_{I}(t)(1-\gamma_{H}) + \sigma_{H}H_{E}(t)$$

$$H_{R}(t+1) =H_{R}(t)(1-\psi_{H}) + \gamma_{H} H_{I}(t)$$

$$V_{O}(t+1) = \mu_{O}V_{O}(t)(1-\nu_{O}) + \sum_{a=1}^{n_{a}} {p_{f}(a)f(a)N}_{V}(a,t)$$

$$V_{L}(t+1) = \mu_{O}\nu_{O}V_{O}(t)+ \mu_{J}V_{J}(t)exp(-\boldsymbol{d}\boldsymbol{V}_{\boldsymbol{J}}\boldsymbol{(t)}\boldsymbol{)}^{\boldsymbol{b}}\boldsymbol{)(1-}\boldsymbol{\nu}_{\boldsymbol{J}}\boldsymbol{)}$$

$${{N^{S}}_{V}}(1,t+1) =(1/2) \nu_{J} \mu_{J}V_{J}(t)exp(-\boldsymbol{d}\boldsymbol{V}_{\boldsymbol{J}}\boldsymbol{(t)}\boldsymbol{)}^{\boldsymbol{b}}\boldsymbol{)}$$

$${{N^{S}}_{V}}(a+1,t+1) =s(a){N^{S}}_{V}(a,t)-s(a)m(a)\lambda_{HV}(t){N^{S}}_{V}(a,t)$$

$${{N^{E}}_{V}}(a+1,t+1) =s(a)m(a)\lambda_{HV}(t){N^{S}}_{V}(a,t)-s(a)\sigma_{V}{N^{E}}_{V}(a,t) + s(a){N^{E}}_{V}(a,t)$$

$${{N^{I}}_{V}}(a+1,t+1) =s(a){{\sigma_{V}N}^{E}}_{V}(a,t) + s(a){N^{I}}_{V}(a,t)$$

a = 1,...,N_A_

**
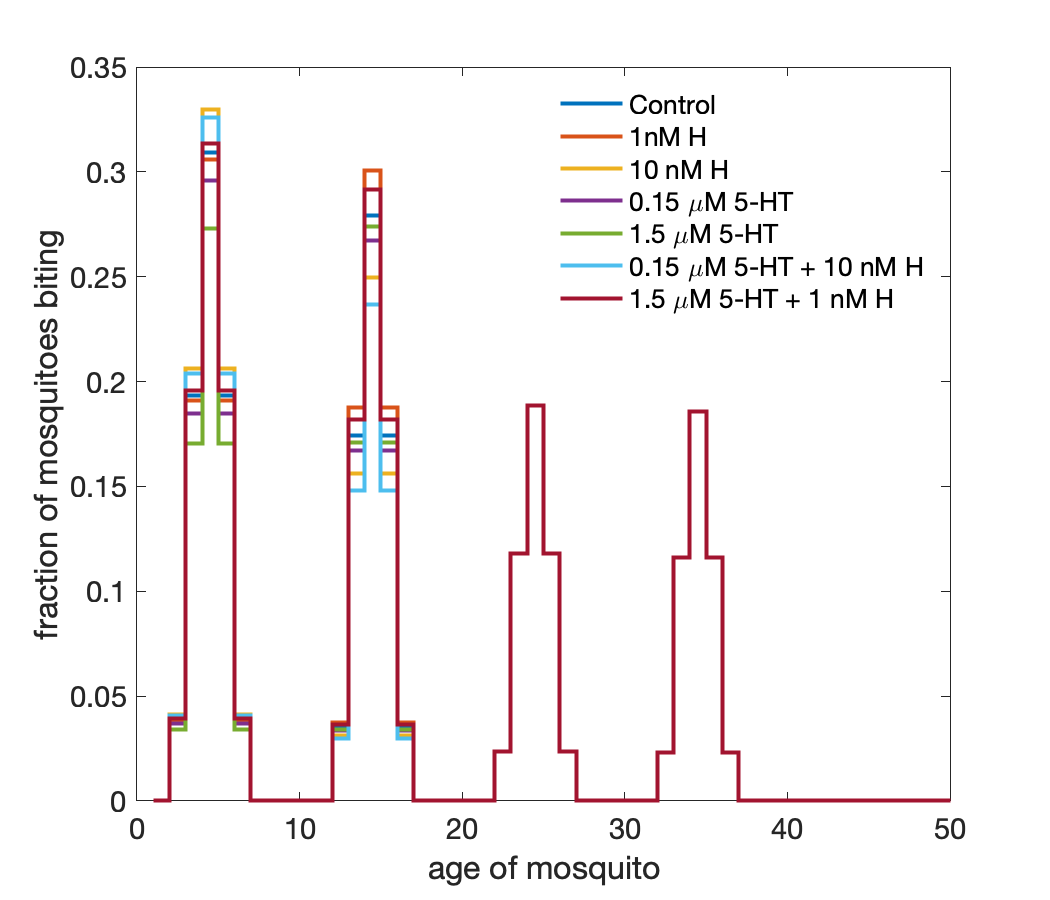
**

**Supplementary Figure 6.** Biting patterns based on treatments. Peaks are set at 4 and 14 days based on experimental from priming experiments. The distribution around the peak divides the total fraction who feed around day *d* across 5 days with 5% biting on days *d*+/-1, 25% on days *d*+\-2, and 40% on day *d*.

**Supplementary Table 4.** Parameters in the model together with descriptions, default values (and range), and source of parameter values. Note that some probabilities are determined by rates, and we assume exponential rates. For these, we have defined the rates here, where the probability *p* is defined in terms of a rate *p_0_* as **p = 1-exp(-p_0_)**. Where the source is marked as *, the values were taken from the present work.

| **Parameter** | **Description** | **Value** | **Source** |
| --- | --- | --- | --- |
| *β_VH_* | The probability of successful transmission of a pathogen from a mosquito to a human upon a successful bite. | 0.3 | Filipe et al. 2007 |
| *β_HV_* | The probability of successful transmission of a pathogen from a human to mosquito upon a successful bite. | 0.25 | Filipe et al. 2007 |
| *c* | The daily biting rate of adult female mosquitoes. This can be thought of as the number of bites a mosquito takes per day. | 2-3 | Detinova et al. 1962 |
| 𝜎_V,0_ | The rate (in days) at which an infected mosquito becomes infectious. This is defined as the inverse of the average extrinsic incubation period. | 1/10 | Thomas et al. 2018 |
| 𝜎_H,0_ | The rate (in days) at which an infected human becomes infectious. This is defined as the inverse of the average intrinsic incubation period. | 1/13 | Collins & Jefferey 1999 |
| γ_H,0_ | The rate (in days) at which a human recovers from being infectious. This is defined as the inverse of the average duration of infection. | 1/200 | Bretscher et al. 2011  Bloland and Williams 2002 |
| ψ_H,0_ | The rate (in days) at which a human loses temporary immunity to malaria. This is defined as the inverse of the average duration of temporary immunity. | 1/300 | Deloron and Chougnet 1992 |
| *μ_1,0_* | The rate daily probability of egg survival. | 0.99 | Assumed |
| *μ_2,0_* | The rate (in days) at which juvenile mosquitoes (larvae and pupae) die. This is defined as the inverse of the average lifespan of juveniles that do not emerge as adults. | 1/14 | Joshi et al. 2014 |
| *ν_1,0_* | The rate (in days) at which eggs hatch. This is defined as the inverse of the average time before hatch. | 1/4.5 | Joshi et al. 2014 |
| *ν_2,0_* | The rate (in days) at which juveniles emerge as adults. This is defined as the inverse of the average time spent as larvae and pupae before emergence. | 1/12 | Joshi et al. 2014 |
| *d,* | Parameters in the density dependence function. Values were chosen to achieve an average mosquito-host ratio. | 1x10^-6^ | Assumed |
| *b* |  | 3.4 | Assumed |
| *VHR* | The vector host ratio, or the number of mosquitoes per human. This value is controlled by demographic parameters. | 10 | Assumed |
| *N_A_* | The largest age class of mosquitoes considered. | 50 | Assumed |
| **p_f_a(a)** | The fraction of females of age *a* who lay eggs. | Varies | * |
| **f(a)** | The number of eggs laid per day by a female of age *a*. | Varies | * |
| **s(a)** | The daily survival probability of a female of age *a*. | Varies | * |

**Model simulation.** For all scenarios, we assume a closed human population of 100,000 people with no previous exposure to malaria, all with homogeneous risk for contact with mosquitoes and for contracting malaria. In short, that means we consider an epidemic scenario where an infected person arrives in an entirely susceptible population. Other than this single arrival of an infectious person, there is no movement in or out of the population during the time frame which we consider.

**Comparing biogenic amine concentration scenarios.** This model has a relatively simple treatment of disease dynamics and mosquito behavior, but we can use this model to investigate different scenarios in which mosquitoes take different concentrations of histamine and 5-HT in bloodmeals by considering their effects on particular life history and behavioral traits. In order to compare the 7 bioamine treatment scenarios in the experimental work, we simulate the model with a parameterization for a scenario given the data generated in the experiments. We then compare each of the scenarios by describing them in relation to the control scenarios. We compare all 7 possible scenarios even when the treatments did not result in significant differences from the control or other treatments.

**Sensitivity to assumptions.** For all parameters not generated from this study, we collected data from literature (Supplementary Table 4). We did, however, make some assumptions about parameters that control the population size of mosquitoes. In particular, the parameters *d* and *b* control density-dependent population regulation. These parameters were chosen to result in a mosquito to host ratio of about 10:1. Below, we show that the qualitative results we present in the main text are not sensitive to the choice of these parameters, although the quantitative values do differ. We plan to explore the mathematical model shown in this work in greater detail in future work.


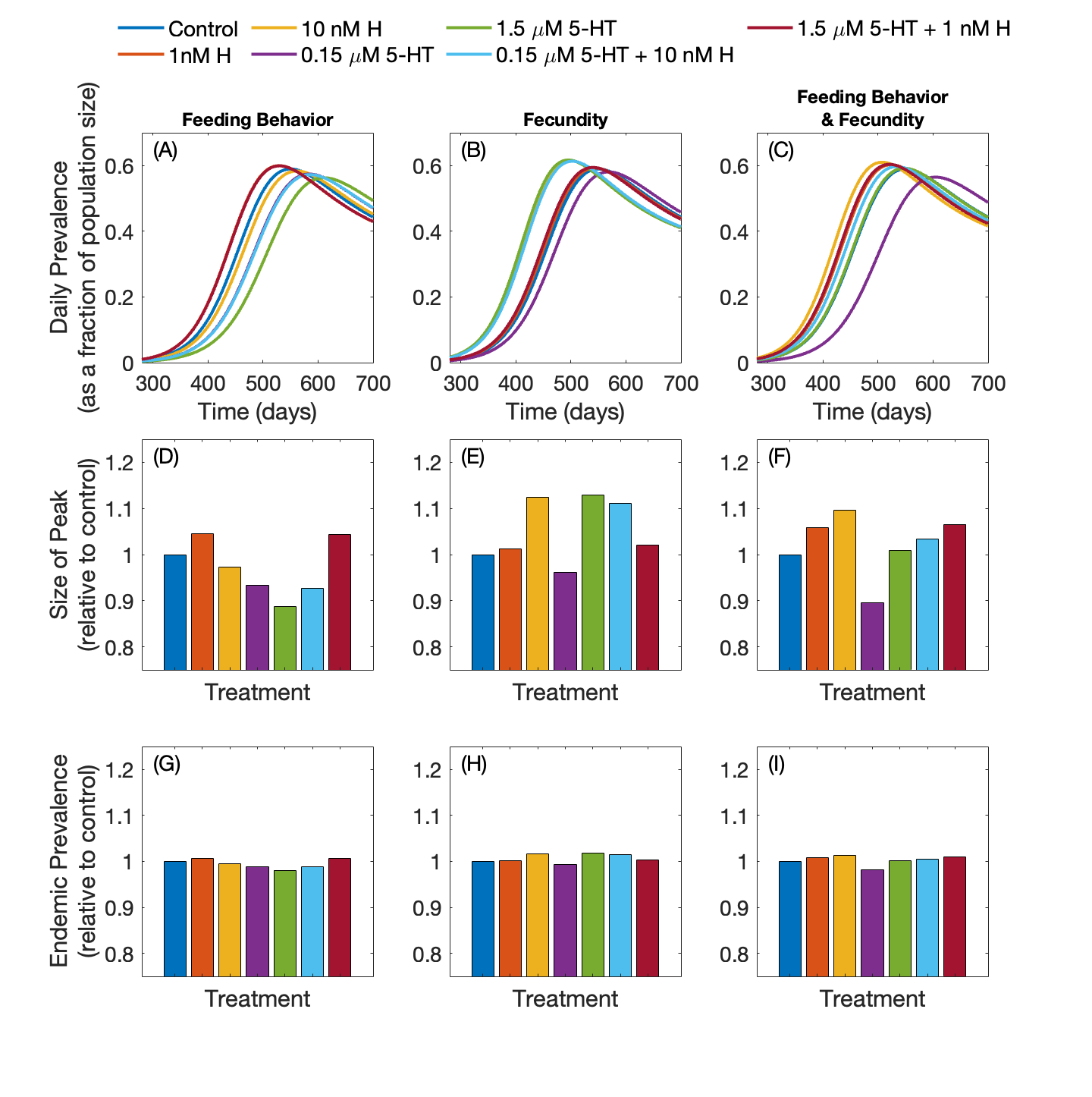


**Supplementary Figure 7.** Results of model simulations corresponding to those of Figure 10 in the main text. Daily prevalence relative to the total population (A-C), size of the peak daily incidence relative to the control (D-F), and endemic prevalence (G-I) for each mosquito group. Effects of mosquito biogenic amines were considered in three scenarios: differences in fecundity (A, D, G), differences in tendency to take bloodmeal at 4 and 14 days (B, E, H), and both changes in fecundity and tendency to take a bloodmeal at 4 and 14 days (C, F, I).


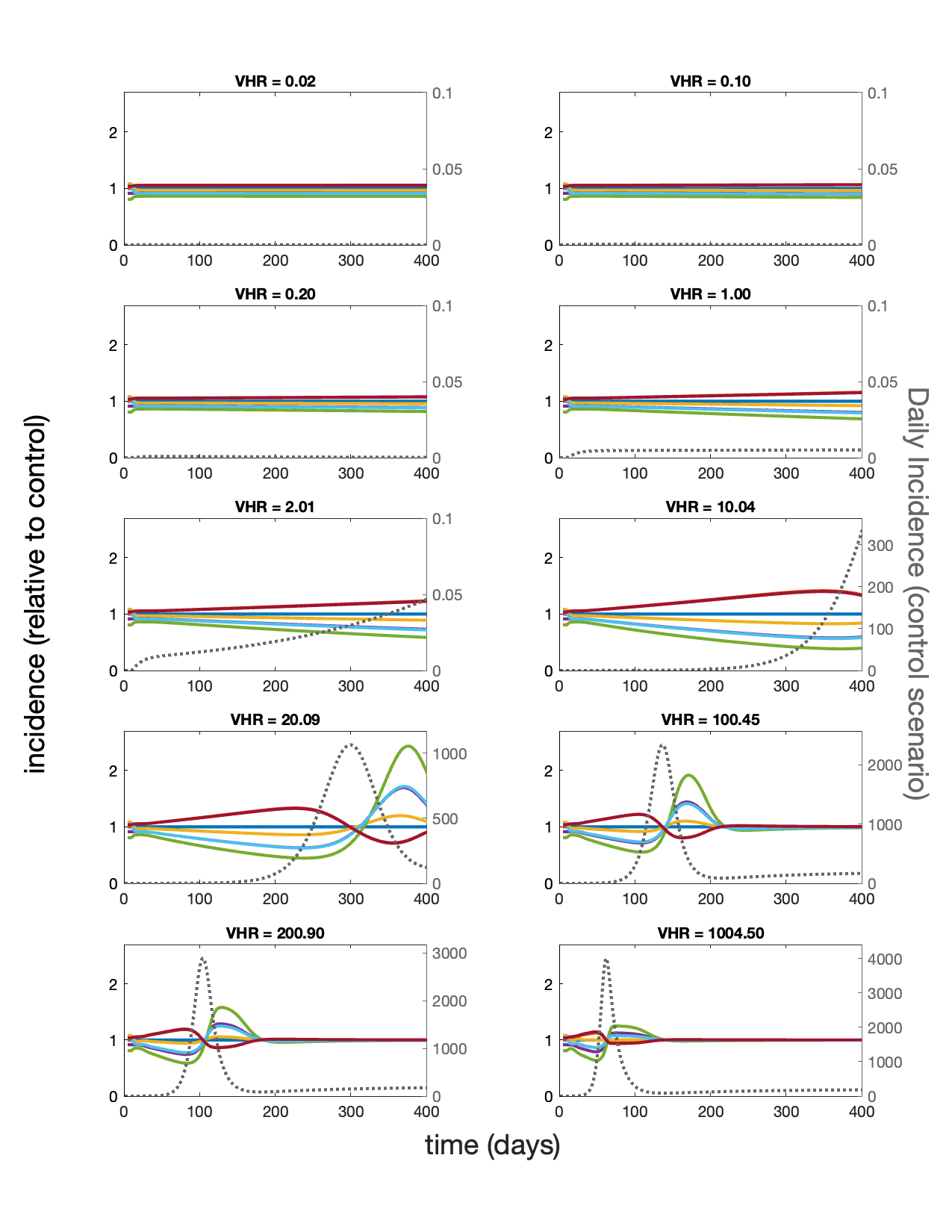


**Supplementary Figure 8.** Sensitivity of model simulations corresponding to those of Figure 10 in the main text for the Feeding Behavior Scenario (**Figs. 10A, D, G**). Each panel shows daily incidence relative to the control (blue) and each of the treatments (colors correspond to those of **Supplementary Fig. 7** and **Fig. 10** in the main text) for different vector-host ratios.

**References**

Bloland, P. B. and Williams, H. A. (2003). Malaria Control During Mass Population Movements and Natural Disasters. *National Research Council, Washington, DC: The National Academies Press.* https://doi.org/10.17226/10539.

Collins, W. E. and Jeffery, G. M. (1999). A retrospective examination of the patterns of recrudescence in patients infected with *Plasmodium falciparum*. *Am. J. Trop. Med. Hyg.* 61, 44-48.

Deloron, P. andChougnet, C. (1992). Is immunity to malaria really short-lived? *Parasitol. Today* 8, 375-378.

Detinova, T. S. (1962). Age-grouping methods in Diptera of medical importance with special reference to some vectors of malaria. Monogr Ser World Health Organ. 47, 13-191. PMID: 13885800.

Filipe, J. A. N., Riley, E. M., Drakeley, C. J., Sutherland, C. J., and Ghani, A. C. (2007). Determination of the process driving the acquisition of immunity to malaria using a mathematical transmission model. *PLoS Comput. Biol.* 3, e255. doi:10.137/journal.pcbi.0030255

Joshi, D., McFadden, M. J., Bevins, D., Zhang, F., and Xi, Z. (2014). Wolbachia strain w AlbB confers both fitness costs and benefit on *Anopheles stephensi*. *Parasit. Vectors*, 7, 1-9.

Thomas, S., Ravishankaran, S., Justin, N. A. J. A., Asokan, A., Kalsingh, T. M. J., Mathai, M. T., Valecha, N., Montgomery, J., Thomas, M. B., Eapen, A. (2018) Microclimate variables of the ambient environment deliver the actual estimates of the extrinsic incubation period of *Plasmodium vivax* and *Plasmodium falciparum*: a study from a malaria-endemic urban setting, Chennai in India. Malar. J. 17, 201. doi: 10.1186/s12936-018-2342-1. PMID: 29769075; PMCID: PMC5956829.
